# Reprocessing of side-streams towards obtaining valuable bacterial metabolites

**DOI:** 10.1007/s00253-023-12458-8

**Published:** 2023-03-16

**Authors:** Kamil Piwowarek, Edyta Lipińska, Marek Kieliszek

**Affiliations:** Department of Food Biotechnology and Microbiology, Institute of Food Sciences, Warsaw University of Life Sciences – SGGW, Nowoursynowska 159C, 02-776 Warsaw, Poland

**Keywords:** Utilisation, Side-streams, Bacterial cellulose, Propionic acid, Vitamin B12, PHAs

## Abstract

**Abstract:**

Every year, all over the world, the industry generates huge amounts of residues. Side-streams are most often used as feed, landfilled, incinerated, or discharged into sewage. These disposal methods are far from perfect. Taking into account the composition of the side-streams, it seems that they should be used as raw materials for further processing, in accordance with the zero-waste policy and sustainable development. The article describes the latest achievements in biotechnology in the context of bacterial reprocessing of residues with the simultaneous acquisition of their metabolites. The article focuses on four metabolites — bacterial cellulose, propionic acid, vitamin B12 and PHAs. Taking into account global trends (e.g. food, packaging, medicine), it seems that in the near future there will be a sharp increase in demand for this type of compounds. In order for their production to be profitable and commercialised, cheap methods of its obtaining must be developed. The article, in addition to obtaining these bacterial metabolites from side-streams, also discusses e.g. factors affecting their production, metabolic pathways and potential and current applications. The presented chapters provide a complete overview of the current knowledge on above metabolites, which can be helpful for the academic and scientific communities and the several industries.

**Key points:**

*• The industry generates millions of tons of organic side-streams each year.*

*• Generated residues burden the natural environment.*

*• A good and cost-effective method of side-streams management seems to be biotechnology – reprocessing with the use of bacteria.*

*• Biotechnological disposal of side-streams gives the opportunity to obtain valuable compounds in cheaper ways: BC, PA, vitmain B12, PHAs.*

## Introduction

According to the Food and Agriculture Organization (FAO), about 1.3 billion tons of food are wasted in the world each year, which corresponds to 30–40% of total annual food production (Gaur et al. 2020). Almost 50% of waste food is fruit, vegetables and root crops. The processing industry, which is a source of post-production residues, is also a significant problem. Ineffective management of side-streams makes them a real global problem. A significant part of organic residues goes to landfill — to the detriment of the natural environment and processing plants, which bear the costs of landfilling. The remaining side-streams are most often used as animal feed, composted or incinerated, discharged into sewage or used as fertiliser (often illegally) (Thi et al. 2015; Waqas et al. 2018). Inappropriate waste management practices can be reflected in problems related to public health and the quality of the environment. Waste incineration is associated with high costs (e.g. infrastructure costs) and huge harm to the environment (air pollution, greenhouse gas emissions). In landfill, organic residues are broken down by microorganisms, the products of their metabolism can contaminate the soil or groundwater. The degradation of organic substances in uncontrolled conditions may be a source of the harmful greenhouse gas methane (Sánchez et al. 2015). Side-stream storage may also lead to the development of rodents and other pests, which are potential vectors of pathogenic microorganisms. It should also be noted that the incineration and storage of waste is associated with unpleasant organoleptic sensations (bad smell) (Waqas et al. 2018).

Taking into account the expected growth of the world population (Take Action for the Sustainable Development Goals – United Nations Sustainable Development. Available online: https: //www.un.org/sustainabledevelopment/sustainable-development-goals/ accessed 09/28/2022), to provide humanity with adequate nutrition, food production must be intensified, which is tantamount to the increased generation of by-products of technological processes or food residues, which, as indicated above, has/will have a negative impact on the quality of our life (Kiran et al. 2014). To avoid this, the contemporary challenge of humanity, industry and science is to reduce food waste, optimise production processes (allowing the minimisation of side-stream production), valorise residues for obtaining valuable products and change the consumption and production model — from a linear type to a closed circuit, the so-called zero-waste policy (Mateo and Maicas 2015). It is important to ensure that all food waste and industrial by-products constitute a resource/raw material for further use.

**Fig. 1:**
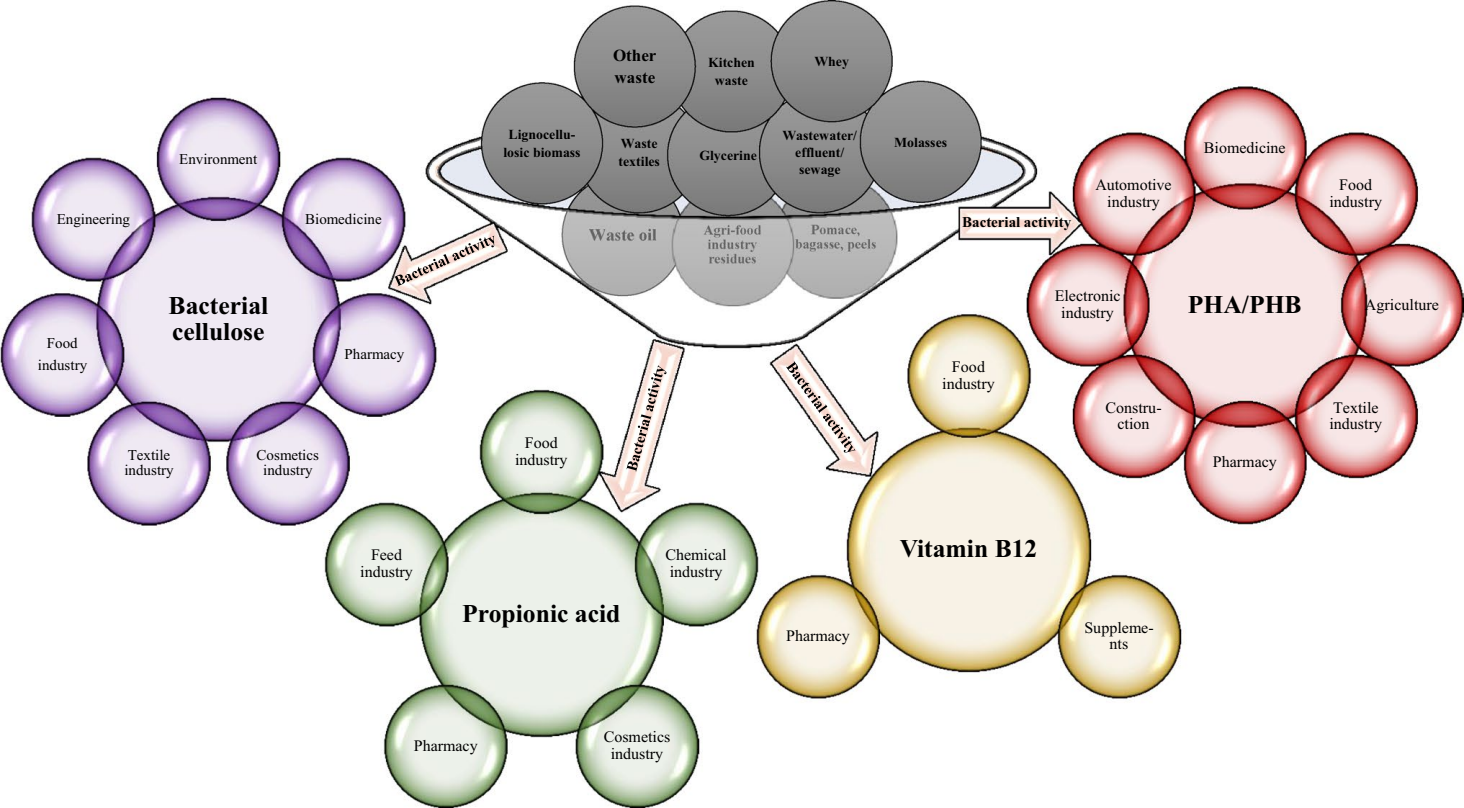
Bacterial reprocessing of side-streams towards obtaining industrially valuable metabolites

Organic residues usually contain a lot of moisture and are often a rich source of biologically active ingredients, i.e. proteins, sugars, minerals, fats or vitamins (Pham et al. 2015). For growth, microorganisms require carbon, nitrogen, enzymatic cofactors and adequate water activity. Taking into account the composition of side-streams and the nutritional needs of microorganisms, it can be concluded that by-products can be an ideal culture media, ensuring the efficient metabolic activity of individual microorganisms, for example, bacteria. In recent years, science has made a huge step towards the commercialisation and popularisation of the idea of recycling organic residues using bacteria. The concept of recovering energy and resources from solid and liquid side-streams is gaining popularity. Not only in the world of science, but also in economic and social areas. The development of this sector of science creates the possibility of bioconverting waste materials into industrially useful compounds. Waste organic matter can be used by microorganisms as a substrate for energy production (in the form of methane, hydrogen or electricity) or for the production of compounds of industrial importance or potential (e.g. organic acids, vitamins, aromas, enzymes, pigments, polyhydroxyalkanoates [PHAs], bacterial cellulose) (Panesar et al. 2015; Rodriguez-Perez et al. 2018; Ali et al. 2021; Urbina et al. 2021; Calvillo et al. 2022; Sharma et al. 2022). Although they can be obtained naturally, some of the above, with the participation of microorganisms, are produced on an industrial scale by chemical means (propionic acid) due to their economic benefits. For other compounds, despite their important functions and applications (food, engineering, biomedical, pharmaceutical industries), global production is small — both chemical and natural — mainly due to the high production costs, regardless of the method (PHAs, bacterial cellulose). Therefore, new innovative solutions that would allow for cheaper production are sought. The solution may be culture media consisting of industrial side-streams. Their utilisation for obtaining propionic acid, bacterial cellulose or PHAs will contribute to the implementation of the zero-waste policy and sustainable development, to the benefit of the environment and the economy. Vitamins, enzymes, flavours and dyes are successfully obtained with the use of microorganisms; however, efforts are still being made to reduce the costs of producing these compounds and, thus, their market price. In the context of vitamins, cobalamin seems to be the greatest challenge, which is derived only from animal foods in the diet. The growing popularity of plant-based diets and ageing society make the problem of cobalamin deficiency a real issue. This means an increase in demand for supplementation with this vitamin, which can be seen in the successive increases in the industrial production of this compound (Calvillo et al. 2022). A common problem limiting the microbiological production of compounds of industrial importance is the economics. The hope for solving this issue is seen in the side-streams, for two reasons: (1) they are a reservoir of compounds assimilated by bacteria; (2) they are cheap and easily available (Yang et al. 2018). Taking into account the fact that the culture medium is most often the largest part of the production costs (Yang et al. 2018; Hussain et al. 2019; Kadier et al. 2021; Piwowarek et al. 2021a), the reprocessing of organic waste materials for acquiring bacterial metabolites may increase the profitability of the microbiological processes for obtaining this type of compound (Fig. 1). The disadvantage of side-streams as culture media may be the fact that some of them require appropriate preparation (pre-treatment) (Tables 1, 2, 3, 4). Some residues require enzymatic or chemical hydrolysis to release carbon sources that can be assimilated and fermented by bacteria (e.g. corn stalk, cassava bagasse, sorghum bagasse, sugarcane molasses, fiber sludges). Other waste materials require yet other methods of pre-treatment, e.g. hot water extraction (necessary to extract nutrients from tchem — e.g. sweet lime pulp, pineapple peel, apple pomace) (Cavka et al. 2013; Wang and Yang 2013; Soemphol et al. 2018; Dubey et al. 2018; Wang et al. 2020; Castro et al. 2021; Piwowarek et al. 2022). It seems, however, that the benefits of pre-treatment far outweigh the disadvantages of such action, which are mainly related to financial outlays and lengthening the process. Low-cost production should contribute to the commercialisation of compounds of microbiological origin, providing cheaper products and displacing chemically synthesised substances from the market (which is not without significance, especially taking into account the social trends related to the consumption/use of products with so-called “clean labels”, and therefore no artificial additives). In addition to obtaining products of natural origin, the use of residues as a culture media creates an alternative for the current methods of managing these kinds of material. The biotechnological utilisation of side-streams may turn out to be an effective way of combating the dangers of producing and collecting residues to the benefit of the environment and public health.

**Table 1:** Reprocessing of different side-streams for bacterial cellulose production

Microorganism	Side-stream(s)	Pre-treatment	Additional mutrient(s)	Culture type	Production	Literature
*Komagataeibacter europaeus* SGP37	Sweet lime pulp Sweet lime pulp	Hot water extraction Hot water extraction	HS medium, glucose –	Static intermittent fed-batch Static batch	38 g/L 6.30 g/L	Dubey et al. 2018
*Gluconacetobacter medellinensis* ID13488	Apple pomace Apple pomace + sugar cane (1.5:2.3)	–	–	Static batch	1.50 g/L 2.50 g/L	Urbina et al. 2017
*Komagataeibacter xylinus* CICC 10529	Citrus peel and pomace	Enzymatic hydrolysis	Yeast extract, ethanol, peptone	Static culture	5.70 g/L	Fan et al. 2016
*Komagataeibacter xylinus* PTCC 1734	Cheese whey + date syrup (50:50)	Date syrup – dilution	Ethanol, vitamin C	Batch fermentation	1.88 g/100 mL	Raiszadeh-Jahromi et al. 2020
*Gluconacetobacter hansenii* PJK (KCTC 10505BP)	Waste from beer fermentation broth Waste from beer fermentation broth Waste from beer fermentation broth Waste from beer fermentation broth	– – Hydrolysis Hydrolysis	Glucose – Glucose -	Flask static cultures Flask static cultures Flask static cultures Flask static cultures	13.95 g/L 8.46 g/L 3.64 g/L 2.82 g/L	Ha et al. 2008
*Komagataeibacter saccharivorans* BC1	Crude distillery effluent	–	–	Flask static cultures	1.24 g/L	Gayathri and Srinikethan 2019
*Komagataeibacter rhaeticus*	Sugarcane molasses	–	Glucose, ethanol, yeast extract	Flask static cultures	4.01 g/L	Machado et al. 2018
*Gluconacetobacter xylinus* ATCC 23770	Fiber sludges: SAFS – from pulp and paper mill using a sulfate-based process SIFS – form lignocellulosic biorefinery using a sulfite-based process	Enzymatic hydrolysis, dilution Enzymatic hydrolysis, dilution	Yeast extract, tryptone	Flask static cultures	11 g/L 10 g/L	Cavka et al. 2013
*Gluconaceter xylinus* BNKC19	Crude glycerol (glycerine) Crude glycerol (glycerine) + pineapple peel extract (50%)	– Pineapple peel extract – hot water extraction	HS medium –	Flask cultures	12.31 g/L > 10 g/L	Soemphol et al. 2018
*Gluconacetobacter xylinus* ATCC 23770	Cotton-based waste textiles	Treated with an ionic liquid (1-allyl-3-methylimidazolium chloride), enzymatic hydrolysis	Peptone, yeast extract	Static cultivation	10.8 g/L	Hong et al. 2012

**Table 2:** Reprocessing of different side-streams for propionic acid production

Microorganism	Side-streams	Pre-treatment	Additional mutrients	Culture type	Production	Literature
*P. freudenreichii*	Whey	–	Casamino acid, casitone, K_3_PO_4_, NaH_2_PO_4_ 2H_2_O, biotin, calcium pantothenate, FeSO_4·_7H_2_O, CoSO_4·_6H_2_O, MnCl_2_·4H_2_O, ZnCl_2_, MgCl_2·_6H_2_O	Flask cultures	22.57 g/L (0.56 g/g)	Kośmider et al. 2010
*A. acidipropionici* ATCC 4875 wild strain *A. acidipropionici* ATCC 4875 mutant strain	Whey Whey	– –	Yeast extract, trypticase Yeast extract, trypticase	Fed-batch fermentation with immobilised cells (PEI-Poraver in FBB) Fed-batch fermentation with immobilised cells (PEI-Poraver in FBB)	125 g/L 135 g/L	Jiang et al. 2015
*P. freudenreichii* CCTCC M207015	Sugarcane molasses Sugarcane molasses + PAB waste cells	Acid hydrolysis Acid hydrolysis	Peptone, yeast extract, NaCl, MnSO_4_, KH_2_PO_4_ –	Fed-bath fermentation in a plant fiber bed bioreactor (PFB) Fed-bath fermentation in a plant fiber bed bioreactor (PFB)	91.89 g/L 79.81 g/L	Feng et al. 2011
*A. acidipropionici* ACT-1	Soy molasses + CSL Soy molasses + CSL Soy molasses + CSL	– Enzymatic hydrolysis –	– – –	Batch fermentation Batch fermentation FBB sequential batch fermentation	21.9 g/L (0.39 g/g) 21.2 g/L (0.46 g/g) 70 g/L (0.42 g/g)	Yang et al. 2018
*A. acidipropionici* ATCC 4875	Corn stover	Deacetylation, dilute acid pre-treatment, enzymatic hydrolysis	Yeast extract	Fed-batch fermentation with high cell density (HCD)	64.7 g/L (0.50 g/g)	Wang et al. 2017
*P. freudenreichii* CICC 10019	Corn stalk	Physical hydrolysis, enzymatic hydrolysis	CSL, potassium dihydrogen phosphate, cobalt chloride	Fed-batch fermentation with in expanded bed adsorption bioreactor (EBAB)	91.4 g/L (0.75 g/g)	Wang et al. 2020
*A. acidipropionici* CIP 53164	Sorghum bagasse	Acid hydrolysis	Tryptic soy broth, MnSO_4_, K_2_HPO_4_, yeast extract	Sequential batch fermentation with immobilised cells	35.3 g/L (0. 61 g/g)	Castro et al. 2021
*P. freudenreichii* DSM 4902	Cassava bagasse, crude glycerol, CSL	Enzymatic hydrolysis	–	Repeated-batch fermentations in the FBB	0.57 g/g	Wang and Yang 2013
*A. jensenii* DSM 20274	Cocoa pod husk	Chemical hydrolysis, enzymatic hydrolysis	Glycerol, yeast extract, mineral salts	Flask cultures	10.58 g/L (0.59 g/g)	Sarmiento-Vásquez et al. 2021
*P. freudenreichii* DSM 20271	Apple pomace, potato wastewater, waste glycerine	Hot water extraction (apple pomace)	–	Flask cultuers	8.15 g/L (0.48 g/g)	Piwowarek et al. 2022

**Table 3:** Reprocessing of different side-streams for vitamin B12 production

Microorganism	Side-streams	Pre-treatment	Additional mutrients	Culture type	_Production	Literature
*P. freudenreichii* DSM 20270	Tofu wastewater	–	Glucose vitamin B2, CoSO_4_·7H_2_O	Flask cultures – anaerobic conditions/LED	10 μg/mL	Yu et al. 2015
*P. freudenreichii* PTCC 1674	Waste frying sunflower oil	Filtration	DMBI, cobalt chloride, iron sulphate, calcium chloride	Flask cultures – aerobic conditions	2.74 mg/L	Haifarajollah et al. 2015
*P. freudenreichii* PTCC 1674	Rice bran oil		Nitrogen sources, DMBI, mineral salts	Flask cultures – anaerobic/aerobic conditions	2.94 mg/L	Hedayati et al. 2020
*P. freudenreichii* CICC 10019	Corn stalk	Physical hydrolysis, enzymatic hydrolysis	CSL, potassium dihydrogen phosphate, cobalt chloride	Fed-batch fermentation with in expanded bed adsorption bioreactor (EBAB)	47.6 mg/L	Wang et al. 2020
*P. freudenreichii*	Glycerine	–	Biotin, Ca pantothenate, CoSO_4_·6H_2_O, NaH_2_PO_4_·2H_2_O, DMBI, casein hydrolysate – depending on the medium variant	Flask cultures – anaerobic/aerobic conditions	4.01 mg/L	Kośmider et al. 2012
*P. freudenreichii* DSM 20271	Apple pomace, potato wastewater	Hot water extraction (apple pomace)	–	Flask cultures – stationary culture	289.80 µg/100 g of wet bacterial biomass	Piwowarek et al. 2022
*P. freudenreichii* DSM 20271	Wheat bran	–	–	Falcon tubes cultures – shaking conditions	155 ng/g of dry wheat bran	Xie et al. 2018
Co-fermentation: *Levilactobacillus brevis* ATCC 14869 *P. freudenreichii* DSM 20271	Wheat bran	–	–	Bioreactor cultures – shaking conditions	332 ng/g of dry wheat bran	Xie et al. 2019
Co-fermentation: *Levilactobacillus brevis* ATCC 14869 *P. freudenreichii* DSM 20271	Rice bran	–	–	Falcon tubes cultures – shaking conditions	742 ng/g of dry rice bran	Xie et al. 2021
*Pseudomonas denitrificans*	Beet molasses	–	Sucrose, betaine, (NH_4_)_2_SO_4_, MgSO_4_·7H_2_O, ZnSO_4_·7H_2_O, CoCl_2_·6H_2_O, DMBI	120 000 L fermenter	181.75 mg/L	Li et al. 2013
*Pseudomonas denitrificans*	Maltose syrup, CSL	–	Betaine, (NH_4_)_2_SO_4_, MgSO_4_, KH_2_PO_4_, ZnSO_4_·7H_2_O, CoCl_2_·6H_2_O, DMBI	120 000 L fermenter	198.27 mg/L	Xia et al. 2015a, b

**Table 4:** Reprocessing of different side-streams for polyhydroxyalkanoates production

Microorganism	Side-streams	Pre-treatment	Additional mutrients	Culture type	Production	Literature
*Cupriavidus taiwanensis*	Corn starch, valerate	–	Na_2_HPO_4_·2H_2_O, KH_2_PO_4_, MgSO_4_·7H_2_O, (NH_4_)_2_SO_4_, (NH_4_)_5_[Fe(C_6_H_4_O7)_2_, CaCl_2_·2H_2_O, trace element was used for the experiment of PHA accumulation	Flask cultures	2.1 g PHA/L	Sheu et al. 2009
*Bacillus megatherium*	Cassava starch	Hydrolysis	H_3_BO_3_, CoCl2·6H_2_O, ZnSO_4_·7H2O, MnCl_2_·4H_2_O, NaMoO_4_·2H_2_O, NiCl_2_·6H_2_O, CuSO_4_·5H_2_O, Na_2_HPO·7H_2_O, KH_2_PO	No mention	29.7% PHB	Krueger et al. 2012
*Photobacterium *TLY01	Soybean oil, corn starch, valerate	–	NaCl, KCl, CaCl_2_⋅2H_2_O, MgSO_4_⋅7 H_2_O, MgCl_2_⋅6 H_2_O, NaHCO_3_, yeast extract, tryptone	Bioreactor	4.01 g PHBV/L	Tian et al. 2022
*Massilia* sp. UMI-21	Starch	–	Na_2_HPO_4_, KH_2_PO_4_, NH_4_Cl, MgSO_4_·7H_2_O, trace element solution	Flask cultures	0.90–1.2 g PHA/L	Han et al. 2014
*Bacillus aryabhattai* T34-N4	Cassava pulp, oil palm trunk starch	–	KH_2_PO_4_, Na_2_HPO_4_, NH_4_Cl, 0.25 g MgSO_4_·7H_2_O, CoCl_2_·6H_2_O, FeCl_3_, CaCl_2_, NiCl_2_.6H_2_O, CrCl_2_.6H_2_O, CuSO_4_·5H_2_O	Flask cultures	17% PHB	Bomrungnok et al. 2020
*Cupriavidus necator* NCIMB 11599	Sugarcane molasses	Filtration, centrifugation	KH_2_PO_4_, (NH_4_)_2_HPO_4_, MgSO_4_·7H_2_O, C_6_H_8_O_7_, trace metal solution contains: FeSO_4_·7H_2_O, CaCl_2_, ZnSO_4_·7H_2_O, MnSO_4_· 4H_2_O, CuSO_4_·5H_2_O, (NH4)_6_Mo_7_O_24_·4H_2_O, Na_2_B_4_O_7_·10H_2_O	Flask cultures	4.60 g P(3HB)/L	Jo et al. 2021
*Cupriavidus necator* 437-540	Sugarcane molasses	Filtration, centrifugation	KH_2_PO_4_, (NH_4_)_2_HPO_4_, MgSO_4_·7H_2_O, C_6_H_8_O_7_, trace metal solution contains: FeSO_4_·7H_2_O, CaCl_2_, ZnSO_4_·7H_2_O, MnSO_4_· 4H_2_O, CuSO_4_·5H_2_O, (NH4)_6_Mo_7_O_24_·4H_2_O, Na_2_B_4_O_7_·10H_2_O	Flask cultures	0.58 g P(3HB-co-LA)/L	Jo et al. 2021
*Cupriavidus necator* Re2058/pCB113	Waste animal fat	–	MSM medium, urea, canola oil	Pilot-scale cultivation 150 L bioreactor	45 g PHA/L	Gutschmann et al. 2023
*Pseudomonas chlororaphis* subsp. *aurantiaca* DSM 19603	Glycerol	–	C_3_H_8_O_3_, (NH_4_)_2_HPO_4_, K_2_HPO_4_, KH_2_PO_4_, MgSO_4_, FeSO_4_·7H_2_O, MnCl_2_·4H_2_O, CoSO_4_·7H_2_O, CaCl_2_·2H_2_O, CuCl_2_·2H_2_O, ZnSO_4_·7H_2_O	Bioreactor cultivation	2.23 g mcl-PHA/L	de Meneses et al. 2020
*Haloferax mediterranei* ATCC 33500	Whey	Hydrolysis	KCl, yeast extract, NaHCO_3_, NaBr, NaCl, CaCl_2_·2H_2_O, MgSO_4_·7H_2_O, MgCl_2_·6H_2_O, ZnSO·7H_2_O, MnCl_2_·4H_2_O, H_3_BO_3_, CoCl_2_·6H_2_O, CuSO_4_, NiCl_2_·6H_2_O, Na_2_MoO_4_·H_2_O	Batch bioreactor	7.92 g P(3HB-co-3HV)/L	Pais et al. 2016

This review discusses biotechnological innovations in the recycling and use of side-streams as substrates for the industrial production of valuable products of bacterial origin — bacterial cellulose, propionic acid, vitamin B12 and PHAs. This article aims to draw attention to obtaining bacterial metabolites of industrial importance from cheap substrates in the form of waste biomass, to the benefit of the economy and the environment. The article presents the most commonly used residues, the current and prospective applications of the above bacterial metabolites, the factors influencing their efficient production, and various ways to improve the production of these compounds. The presented chapters provide a complete overview of the current knowledge on bacterial cellulose, propionic acid, vitamin B12 and PHAs, which can be helpful, among others, for the academic and scientific communities and the agri-food, biotechnology and biomedical industries.

## Bacterial cellulose

Cellulose is the most abundant polymer on Earth. It is produced by plants (mainly) and some microorganisms–microbial/bacterial cellulose (BC). Microbial cellulose is synthesised by algae, fungi and bacteria (Gram-negative, strictly aerobic). Cellulose-producing microorganisms are isolated from vinegar, fruits, vegetables, fermented juices or alcoholic beverages (Urbina et al. 2021).

BC, depending on the species/type of bacteria, is synthesised by various metabolic pathways and differs in its nanostructure, physicochemical and crystalline properties. Bacteria belonging to the Enterobacteriaceae family, *Salmonella typhimurium* and *Escherichia coli*, synthesise amorphous cellulose, while other strains, which constitute the vast majority, produce crystalline cellulose (Zogaj et al. 2001) — *Agrobacterium*, *Sarcina*, *Rhizobium*, *Azotobacter* and *Komagataeibacter* (Ullah et al. 2016, 2017; Tsouko et al. 2015; Ul-Islam et al. 2020). The most efficient production of BC is characterised by species belonging to the acetic acid bacteria (*Komagataeibacter xylinus*, formerly *Acetobacter xylinus*, *Acetobacter xylinum*, *Gluconacetobacter xylinus*) (Yamada et al. 2012; Hussain et al. 2019; Urbina et al. 2021). The mentioned species is a model microorganism, used in both laboratory research and commercially. In addition to the high production efficiency of BC, it also has the ability to use a variety of carbon sources (Lin et al. 2020). Acetic acid bacteria have been used in industry for years (for the production of vinegar, wine vinegar, apple cider vinegar); they are safe, some have GRAS status (generally recognised as safe) and produce BC with high yields. All this makes them the only group of microorganisms which can be used to obtain BC on an industrial scale.

## Properties and applications of BC

Plant cellulose and BC have the same molecular formula (C6H10O5)n, the same chemical structure (linear homopolymers of D-glucopyranose residues linked by β-(1 → 4)-glycosidic bonds), but differ in their physicochemical properties. BC is distinguished by higher purity (no hemicellulose and lignin), higher water absorption, higher hydrophilicity (hydrogel with high water retention capacity – 100 times its weight), higher mechanical strength, better crystallinity (> 80%, while plant cellulose contains more amorphous areas with crystallinity in the 40–85% range), higher porosity and a higher degree of polymerisation. In addition, BC shows no toxicity, has tensile strength, resistance to thermal or chemical shock, biodegradability (hydrophilic materials are more susceptible to hydrolysis, a process which initiates polymer degradation and allows the material to be colonised by microorganisms and fungi that will have access to carbon as food) and is easy to sterilise (Eslahi et al. 2020; Torgbo and Sukyai 2020; Urbina et al. 2021). Pure BC lacks some important features that are important in the application context. It does not exhibit antimicrobial activity, magnetic, conductive or biocompatible properties. However, these obstacles can be overcome by creating BC composites in combination with materials or compounds with the appropriate properties. Literature data show that BC can be surface, chemically or structurally modified (Ul-Islam et al. 2012; Badshah et al. 2018; Kadier et al. 2021) in ex situ (BC is modified after synthesising by methods such as impregnation, immersion, oxidative polymerisation, ultraviolet-induced polymerisation) and in situ way (modifying materials are added to the culture medium during fermentation, so that they are incorporated into the network of BC fibrils) (Andriani et al. 2020).

**Fig. 2:**
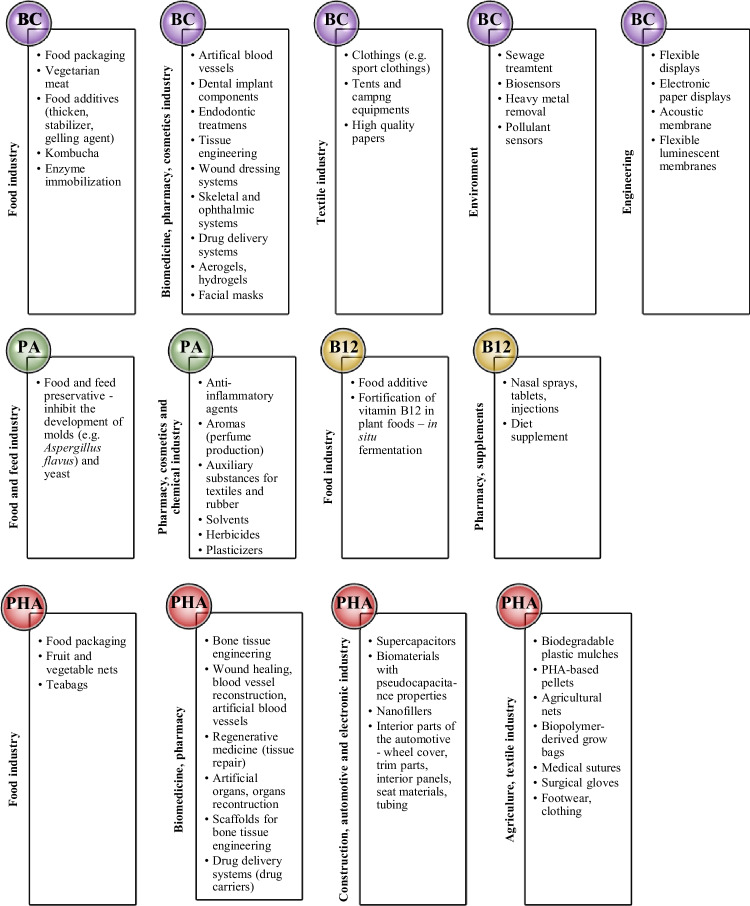
Industrial applications of bacterial metabolites

BC membranes have free hydroxyl groups on the surface; therefore, a given polymer can be easily modified with other polymers or additives to obtain the special properties of this metabolite (Sun et al. 2019). An example of in situ modification is the study by Liyaskin et al. (2018), who used the addition of alginate during the cultivation of bacteria of the species *Komagataeibacter sucrofermentans* B-11267. As a result of cultivation (5 days, 28 °C), they obtained a BC/alginate nanocomposite, which showed biotic activity against bacteria of the *Staphylococcus aureus* species. The researchers indicated wound dressings as a potential application. In the context of ex situ modification, the following were used as modifying substances: collagen, chitosan, 2-acrylamido-2-methylpropane sulfonic acid (AMPS) and multi-walled carbon nanotubes (MWCNTs). The first two cause the cellulose to gain greater biocompatibility compared to pure BC (potential applications: wound dressings, scaffolds for tissue engineering), while the other compounds improve the transparency and conductivity of BC (potential applications: fuel cells, biological systems: artificial muscles, artificial blood vessels) (Cai et al. 2009; Kim et al. 2011; Kadier et al. 2021). BC can be used in many industries: biomedical, pharmaceutical, cosmetic, food (BC has GRAS status, it can be used in food production), textiles, engineering and environmental protection (Khalid et al. 2017; Ullah et al. 2017; Naseri-Nosar and Ziora 2018; Hussain et al. 2019; Kadier et al. 2021) (Fig. 2).

## BC synthesis

*Komagataeibacter xylinus* bacteria produce BC in multiple stages. When glucose is used as a carbon source, the BC synthesis pathway consists of four enzymatic steps: (1) glucose phosphorylation (glucokinase); (2) isomerisation of glucose-6-phosphate (Glc-6-P) to glucose-1-phosphate (Glc-1-P) with the participation of phosphoglucomutase; (3) synthesis of UDP-glucose (UDPGlc) with the use of UDPG-pyrophosphorylase (UGPase); and (4) production of cellulose (cellulose synthase reaction). If disaccharides are used for the synthesis of BC, for example, sucrose, the production of cellulose begins with the hydrolysis of a given carbon source to monosaccharides (glucose, fructose). The first three enzymatic reactions lead to the production of uridine diphosphoglucose (UDPGlc), a direct precursor to BC synthesis. The last step is the synthesis of BC and its release into the medium. An important role in the synthesis of BC is played by cyclic diguanylic acid (c-di-GMP), which is an allosteric activator for cellulose synthase. In the absence of c-di-GMP, cellulose synthase remains inactive or shows low enzymatic activity, resulting in the absence or a reduced yield of synthesised BC. Cellulose particles are synthesised inside the bacteria, then they are spun (during excretion into the medium through the pores) into protofibrils (single cellulose chains) with a diameter in the range of 2–4 nm. The protofibrils combine to form monofilaments, which then aggregate. A ribbon-shaped microfibril with a length of about 80 nm is assembled from the protofibrils. The generated BC contains surface hydroxyl groups that form numerous inter- and intrafibrillar hydrogen bonds, hence, the mechanical strength of BC, its hydrophilicity and susceptibility to chemical modifications (Lee et al. 2014; Urbina et al. 2017, 2021).

Bacteria produce cellulose in the log phase of growth and in the stationary phase. For efficient BC synthesis, they need oxygen, carbon, nitrogen, iron, zinc and vitamins (Hussain et al. 2019). BC production can take place in two ways (depending on the desired microstructure and properties of BC): (1) a static culture and (2) a shake culture. With a static culture (a simple technique often used to produce BC on a laboratory scale), the BC is synthesised at the air/liquid interface. The microbial cells are trapped in a polymer network that provides the bacteria with protection (biological, chemical, mechanical) and keeps the microbes close to the air. The thickness of the BC depends on the incubation time. Usually cultivation lasts 14 days, extending it for another hours (longer fermentation time) results in the accumulation of compounds that inhibit bacterial metabolism (glycolic acid, formic acid) (Kiziltas et al. 2015; Urbina et al. 2021). BC obtained by static cultivation is characterised by a rigid and strong structure, high modulus of elasticity, tensile strength, strong ability to hold its shape and tear resistance (potential applications: food packaging, scaffolding in biomedicine, synthetic blood vessels) (Gao et al. 2019). The advantage of this type of culture is that the process does not produce mutants that do not have the ability to synthesise BC. The disadvantages of static culture are: high production costs, long fermentation time and low productivity.

BC from a shake culture (shaking provides aerobic conditions) is in the form of a fibrous suspension, spheres, granules. The shape and size depend on the time of cultivation and the speed of mixing. The production of BC in a shake culture takes place throughout the medium and is not limited to the air/liquid interface, oxygen and nutrients are dispersed throughout the volume of the medium. A shake culture, compared to BC from a static culture, provides BC which is characterised by a less ordered structure, lower crystallinity and higher hygroscopicity (Urbina et al. 2021). These features predispose BC from a shake culture for use in drug delivery systems (Hoshi et al. 2018). In the context of industrial production, the shaking method is more effective compared to static breeding. The main disadvantage of this technique are mutants arising during cultivation that do not produce cellulose, which reduces the efficiency and productivity of this type of polymer synthesis (Hsieh et al. 2016). Finally, regardless of the method, BC undergoes purification (the removal of non-cellulosic compounds). For this purpose, it is rinsed with a solution of NaOH or KOH, resulting in a smooth gelatinous product (approx. 99% of water content) (Urbina et al. 2021).

The main factor limiting the industrial synthesis of BC is the profitability of the production process. One of the problems is the low productivity and efficiency of microbial cellulose synthesis. In order to intensify the production of BC, various bioreactors have been developed: a modified airlift bioreactor (an internal loop airlift with enriched oxygen, an internal loop airlift with controlled pH and a carbon source), a biofilm bioreactor, a rotating disc reactor, a rotary biofilm contactor (microorganisms are not exposed to shear stresses and can easily take oxygen from the air; using this type of reactor with *Gluconacetobacter* sp. produced 5.67 g BC/L) (Kim et al. 2007), a bioreactor equipped with a spin filter and a reactor with a silicone membrane (Lin et al. 2014; Ul-Islam et al. 2017; Andriani et al. 2020; Kadier et al. 2021). Cheng et al. (2009) used corn steep liquor with fructose (CSL-Fru) and supplements for the production of BC; the process was carried out in a bioreactor with a plastic composite carrier (PCS; polypropylene acts as a matrix that integrates the substrates e.g. ground husks or microbial nutrients — peptone, yeast extract; PCS provides the appropriate structure for biofilm formation and proper release of nutrients during cultivation). The use of a PCS allowed 7.05 g/L of BC to be produced (2.5 times more than in the control, no PCS) while maintaining high crystallinity (93%), crystal size (5.2 nm), strength and tensile strength. The advantages of bioreactor cultures are: the possibility of using static and shake cultures, fast oxygen transfer rate, high fermentation volume efficiency, higher productivity, easier control of culture conditions and industrial production. Considering the above, it should be borne in mind that the choice of fermentation method depends on the physical, morphological and mechanical properties expected from the BC.

## Factors influencing BC synthesis

To optimise the production of BC, bacteria should be provided with appropriate culture conditions (temperature, pH, oxygen supply). The optimal temperature of BC synthesis is in the 25–35 °C range, while the pH is 4.0–7.0 (Urbina et al. 2017, 2021). The culture temperature has been shown to influence the morphology and crystal structure of the BC (Zeng et al. 2011). It should be noted, however, that the optimal temperature or pH of the culture depend on the bacterial strain used. Castro et al. (2012) isolated a strain of *Gluconacetobacter medellensis*, which has the ability to synthesise BC at a pH of 3.5. It should also be remembered that the pH of the medium during cultivation may change due to the accumulation of secondary metabolites (gluconic, acetic, formic and lactic acids). Therefore, in order to enhance the synthesis of BC, the pH of the culture medium should be kept at a level that ensures the maximum yield of BC. The addition of CSL (corn steep liquor) as a buffering substance can be used, for instance (Noro et al. 2004). Both the production efficiency and quality of the BC depend on the dissolved oxygen content in the culture medium. On the other hand, if the concentration of oxygen in the medium is too high, it intensifies the production of gluconic acid, which in turn negatively affects the viability of cells and, thus, the synthesis of BC (Tantratian et al. 2005). This shows that during the production of BC, the medium should be monitored for dissolved oxygen concentration, which should be between 10 and 15% (de Andrade Arruda Fernandes et al. 2020).

The most commonly used medium for the synthesis of BC is the HS medium (Hestrin and Schramm), consisting of D-glucose, peptone, yeast extract, dibasic sodium phosphate and citric acid. Literature data show that many other media have been used for the production of BC: Hassid-Barker (HB), Yamanaka, Zhou, Son, Park, M1A05P5, CSL-fructose, yeast extract-peptone-dextrose (YPD), acetate buffered medium (AB), modified HS medium (MHS) (Hussain et al. 2019).

One of the most important components of the medium in terms of the efficiency of BC synthesis is the carbon source. Many different potential carbon sources have been tested: monosaccharides (glucose, fructose), oligosaccharides (sucrose, galactose), organic acids (citrate, succinate and gluconate), alcohols (ethanol) and sugar alcohols (D-mannitol, D-arabitol, glycerol) (Lee et al. 2014; Mohammadkazemi et al. 2015; Urbina et al. 2021). Studies have shown that the preferred carbon sources for BC production by *Acetobacter xylinum* bacteria are D-arabitol and D-mannitol; fermentation of these carbon sources resulted in, respectively, 6.2- and 3.8-fold higher production rates of BC compared to glucose (Jonas and Farah 1998). Pourramezan et al. (2009) found that sucrose is the sugar that guarantees the most efficient synthesis of BC, followed by glucose, xylose and lactose. When using glucose, the appropriate concentration should be selected. The higher the glucose concentration in the medium, the lower the BC production. The use of glucose as a carbon source results in the production of gluconic acid (a by-product) which lowers the pH of the medium, which in turn inhibits the production of BC (Lee et al. 2014). A positive effect on the synthesis of BC was found when using of ethanol as a supplement. Son et al. (2001) used the addition of ethanol at 1.4% (v/v) during cultivation of the *Acetobacter* sp. A9 strain, which increased the BC production efficiency by 400% (15.2 g/L) in comparison with the control medium (without the addition of ethanol). There are two reasons for this: (1) ethanol suppresses the spontaneous mutation of cellulose-producing bacteria into mutants without the ability to synthesise this substance (a particular problem in shake cultures) (Wang et al. 2019); (2) ethanol is an additional carbon source enhancing the kinetics of BC synthesis. Ethanol intensifies the synthesis of ATP which, in the pathway of bacterial cellulose synthesis, enhances the activity of glucokinase and fructokinase, and inhibits the activity of gluconokinase and glucose 6-phosphate dehydrogenase in pentose phosphate metabolism (Wang et al. 2018), resulting in an increase in the efficiency of BC production, but, importantly, only with the use of the right concentration of ethanol (Lee et al. 2014; Mohammadkazemi et al. 2015).

In terms of the synthesis of BC, nitrogen sources are also important and are necessary in the context of building cellular biomass, for example, peptone, yeast extract, glycine, casein hydrolysate, ammonium sulphate, glycine and tea extracts (green, black, silk) (Ul-Islam et al. 2017; de Andrade Arruda Fernandes et al. 2020). The media for the cultivation of acetic acid bacteria for BC synthesis were also supplemented with disodium phosphate, magnesium, sulphur and potassium salts (de Andrade Arruda Fernandes et al. 2020).

Vitamins that stimulate the synthesis of BC are vitamin C (reduces the production of gluconic acid through antioxidant activity), pyridoxine, nicotinic acid, p-aminobenzoic acid and biotin (enhances the synthesis of BC by intensifying the yield of bacterial biomass), while pantothenate and riboflavin limit the production of cellulose (Keshk 2014; Lee et al. 2014). In addition to the above, to enhance the kinetics of BC synthesis, the media was also supplemented with: acetic acid (a fourfold increase in production), CaCO_3_ (a twofold increase in production, from 3.7 to 7.4 g/L), lactic acid (the production of BC increased by 4–5 times), amino acids (a tenfold increase in production) (Rani and Appaiah 2013).

The effect of genetically modifying cellulose-producing bacteria on the increase in the production efficiency of this metabolite was also investigated. Various mutagenic factors were used, for example, UV radiation, high hydrostatic pressure, N-methyl-N-nitro-N-nitrosoguanidine and UV radiation combined with the action of ethyl methanesulfonate. The applied techniques increased the efficiency of BC synthesis (Andriani et al. 2020). Kuo et al. (2015) genetically modified the *Gluconacetobacter xylinus* bacterial strain by knocking-out the membrane-bound GDH gene via homologous recombination of a defect GDH gene. Glucose dehydrogenase (GDH) oxidises glucose to gluconic acid, which limits the conversion of glucose to BC, resulting in a lower yield of this metabolite. Research by Kuo et al. (2015) showed that the production of BC using mutant GDH-KO was approx. 40% (static culture) and 230% (shake culture) higher compared to the wild-type strain.

Literature data show that in order to increase the efficiency of BC synthesis, and thus the profitability of the industrial production of this metabolite, co-fermentation of acetic acid bacteria with other microorganisms was used (Seto et al. 2006; Liu and Catchmark 2019). Seto et al. (2006) used bacteria of the species *G. xylinus* and *Lactobacillus mali* in a CSL (4% w/v)/sucrose medium (4% w/v). Co-fermentation of the above strains increased the cellulose production efficiency threefold in comparison with the acetic acid bacteria monoculture. Liu and Catchmark (2019) conducted the co-fermentation of *G. hansenii* ATCC 23769 and *E. coli* bacteria (ATCC 700728) in an HS medium under static conditions. As a result of co-fermentation, a higher volumetric yield of BC synthesis was obtained (10.8% more BC dry matter was formed – 1.56 g/L) compared to the *G. hansenii* monoculture.

## BC production from side-streams

The constant increase in consumption contributes to the generation of more and more residues. In recent years, a lot more attention has been paid to sustainable development and the zero-waste approach. The goal of the concept mentioned is to keep side-streams in the economic cycle for as long as possible. One possibility for the disposal of waste materials is their use as microbiological media. Recycling of residues by using safe microorganisms with the simultaneous acquisition of the metabolites of these microorganisms seems to be an extremely promising concept. Attention should be paid to the search for such waste materials that would guarantee the richness of various nutrients (sugars, amino acids, vitamins, minerals). Thus, they would be an alternative to conventional, often expensive, laboratory materials (sources of carbon, nitrogen, vitamin nutrients). The most important element limiting the production of some compounds on an industrial scale (BC, propionic acid, PHA/PHB), and thus their applicability, are the costs of the media, which in the case of BC synthesis, it constitutes up to 65% of the total cost (Kadier et al. 2021). This is due to the high prices of the pure laboratory substrates (sources of carbon, nitrogen, mineral salts, vitamins) necessary to create a medium that guarantees efficient yields of the metabolites. Hence, to develop less expensive media, in recent years, scientists have searched for alternative nutritional sources, for example, in the form of residues (cheap, easily available), which would favour the metabolic activity of bacteria (waste recycling through fermentation). Cheaper production will contribute to the industrialisation and commercialisation of bacterial metabolites, and provide a cheaper end product which will be more widely used in various industries.

The microbiological disposal of waste and the by-products of technological processes, with the simultaneous collection of BC, will help in waste management and preventing environmental degradation, as well as contributing to sustainable development and responsible production and consumption. The following materials have been used for the production of BC: waste from the alcohol and non-alcoholic beverages industry, agro-industrial side-streams, textile residues, waste from biodiesel production, lignocellulosic waste, side-streams from the sugar industry and food waste (Hussain et al. 2019; Kadier et al. 2021) (Table 1).

The beverage production sector is one of the largest food processing industries. The production of beverages, both alcoholic and non-alcoholic, results in a huge number of side-streams (including pomace, peel, sewage), which are an abundant source of biologically active compounds, such as: sugars, proteins, organic acids, vitamins and microelements. The situation is similar for agro-industrial waste which, due to its composition, easy availability and low market value, is a potential source of energy for fermentation purposes. The agri-food industry produces huge amounts of waste biomass daily, of which only a small part is further used, most often in the form of animal feed. The European food market generates around 250 million tons of by-products annually. The residues from fruit and vegetable processing account for approx. 10%, of which 30–50% of this residue comes from pressing, for example, juices (Szymańska-Chargot et al. 2017). Fruit pomace alone reaches millions of metric tons (MMT) per year (Krivokapić et al. 2021). The pomace recovered for further use is only 20%. The rest is landfilled, composted or incinerated. In the United States of America (USA), the storage fees for apple pomace alone exceed $10 million annually (Shalini and Gupta 2010; Magyar et al. 2016). Some waste materials, due to the high water content and rich composition, show low microbiological stability and are susceptible to spoilage, which lowers their technological value and poses a risk of environmental contamination. Some of the residue is preserved where it is generated (e.g. by drying, freezing, pickling). Such a solution prevents environmental degradation and enables its further use, for example, in the form of animal feed. Importantly, not all processing plants can afford to preserve the side-streams, this option is associated with investment (appropriate equipment) and operating costs (energy). For small industrial plants, especially at the beginning of their operations, this is often an unattainable financial ceiling. It seems that a good way of managing waste materials (unfixed) is biotechnological reprocessing with the participation of microorganisms (e.g. bacteria which are a source of metabolites of industrial importance), to the benefit of the environment and the economy of production plants. Therefore, in order to valorise by-products from the production of beverages or the agri-food industry, the possibility of producing BC using various residues (e.g. apple pomace, grape pomace/bagasse, citrus pomace, whey, potato wastewater, crude distillery effluent, waste from beer fermentation broth) from these industrial sectors has been examined recently.

Taking into consideration the global processing of apples, it is assumed that several million tons of apple pomace are produced annually across the whole world. For example, the USA generates about 1 million tons of apple pomace annually, whereas in Brazil and Germany it is, respectively, 800 thousand and 250 thousand tons (Magyar et al. 2016). In Poland (the largest producer of apples in the European Union), the production of pomace is in the range of 400–600 thousand tons/year. Apple pomace contains sugars (glucose, fructose), B vitamins and micro- and macro-elements (Magyar et al. 2016; Piwowarek et al. 2022). Their composition suggests that they can serve as a culture medium. Urbina et al. (2017) used apple pomace from cider industry for the production of BC. Scientists cultivated (static culture, 28 °C, 14 days) *G. medellinensis* ID13488 in six variants of media: (1) only pomace, (2–5) different pomace proportions + different sugarcane proportions, (6) HS medium (control). In the medium consisting only of apple pomace, the tested strain produced 1.5 g BC/L, while in the HS medium it was 1.8 g/L. The highest volume yield of BC synthesis (2.5 g/L) was obtained in the medium with apple pomace and sugarcane in the ratio of 1.5/2.3. The following properties of the BC membranes obtained were checked: microstructure, crystallinity, water retention capacity. It was found that BC obtained from pomace and sugarcane had better properties compared to BC obtained from the HS medium.

The global annual grape production is around 50 million tons, of which 75% is used by the wine industry. As a result of processing, residues consisting of peel, seeds and stalks, are generated, which is collectively referred to as pomace which constitutes up to 25% of the weight of the raw material used in the vinification process. The greatest amount of grape pomace (1200 tons per year) is produced in wine-growing countries, that is, Italy, France and Spain (Beres et al. 2017). A policy to reduce post-production side-streams is needed to achieve a sustainable wine production process. Vazquez et al. (2013) investigated the possibility of producing BC by bacteria of the species *Gluconacetobacter xylinus* in media containing grape bagasse (waste from wine production) or bagasse with CSL (a by-product of wet corn milling, a nitrogen source). For comparison, Vazquez et al. (2013) also cultivated the same strain in the HS medium with the addition of various carbon sources (glucose and cane molasses). Regardless of the medium variant, cultures were grown for 14 days under static conditions at 28 °C. The greatest amount of BC was formed in the medium with bagasse and CSL (8.00 g/L). It is also worth noting that the production of BC from grape bagasse only (4.20 g/L) was two times higher compared to the HS medium with glucose as the carbon source. Using bagasse and CSL contributed to improving the economy of BC synthesis. A medium consisting only of these two substrates, without any additional supplementation, increased the productivity of the process, as well as using a cheaper substrate rather than more expensive ones such as yeast extract. The BC ribbons obtained from the side-streams showed the values of width (35–70 nm) and thickness (13–24 nm) similar to the dimensions of the cross-sections of the BC ribbons obtained by culturing *G. xylinus* in the HS medium. The crystallinity of cellulose produced from wine residues (74%) was also similar to BC synthesised in the HS medium (77%). Grape bagasse is an attractive source of nutrients, allowing a reduction in the costs of industrial production of BC.

World production of bananas is around 102 million tons per year. The processing of bananas produces side-streams in the form of leaves, stalks and peels. The largest part is the peel — 35% of the weight of the fruit, which is about 36 million tons of banana peel per year. The most common methods of disposing of this side-stream are landfill and incineration, which causes major environmental problems (Pathak et al. 2016). Taking into account the tonnage of this waste material and its composition (sucrose, glucose, fructose, cellulose, hemicellulose, minerals) (Pathak et al. 2016), it seems that banana peel can be an interesting substrate in biotechnological processes. Khami et al. (2014) fermented banana peels as a carbon source for *A. xylinum* bacteria. The tested material was mixed with the HS medium. After 15 days of cultivation at 30 °C, they obtained 19.46 g BC/L. The characteristics of the cellulose obtained showed potential application as a biopolymer.

Annually, around 100 million tons of citrus are produced worldwide, of which 25–30% goes to processing, which generates huge amounts of residues—pulp, peels and seeds. Citrus side-streams are a rich source of sugars (glucose, fructose, sucrose), fibre (pectin, xylan), organic acids (citric acid, abelic acid), vitamins (ascorbic acid), minerals (Ca, K) and amino acids (Sharma et al. 2017). This suggests that this material can act as a cheap culture medium. Dubey et al. (2018), for the purpose of BC synthesis, cultivated (static conditions, 30 °C, 16 days) the *Komagataeibacter europaeus* SGP37 strain with the simultaneous utilisation of sweet lime pulp waste. They used several variants of the culture media: sweet lime pulp (SLPW), SLPW + HS (no glucose), 50% SLPW + HS (no glucose), 25% SLPW + HS (no glucose), SLPW + HS (with glucose). They also used two variants of SLPW — non-hydrolysed and acid-hydrolysed. The highest production of BC was achieved in the SLWP + HS (with glucose) medium in the variant without hydrolysis (26.2 g/L). In the medium which consisted only of SLPW, the volumetric yield of BC synthesis was 6.30 g/L — for the variant without hydrolysis. Changing the culture strategy (30 °C, 16 days) for the SLPW + HS medium (with glucose) without hydrolysis from static batch to static intermittent fed-batch (IFB-48 h) increased the efficiency of polymer synthesis to 38 g/L (one of the highest from all studies to date). The culture technique used opens up new possibilities for the valorisation of other industrial side-streams. The more so as, according to Dubey et al. (2018), the BC obtained from SLPW, in terms of physicochemical properties, was similar or even better than cellulose obtained from the HS medium. Fan et al. (2016) used the *Komagataeibacter xylinus* CICC 10529 strain and citrus peel and pomace supplemented with yeast extract, peptone and ethanol for the production of BC. The production efficiency of BC (static culture, 30 °C, 8 days) from the side-stream medium was 5.7 g/L, while from the control medium (HS medium) it was 3.9 g/L. The average diameters of the BC fibres obtained from the side-stream medium and the HS medium were 50 and 60 nm, respectively. The crystallinity index of BC from citrus peel and pomace was about 63%, while it was 65% from the HS medium. There were no significant differences in the colour parameters of the tested BCs.

Raiszadeh-Jahromi et al. (2020) made an attempt to optimise the production of BC with cheese whey and date syrup — for this purpose, they cultivated *K. xylinus* PTCC 1734 (28 °C, 10 days) bacteria in various variants of media, with different percentages of individual waste materials. Moreover, they checked the effect of vitamin C supplementation on BC synthesis (they used different doses: 0%, 0.01%, 0.04%). The maximum production of BC (18.8 g/L) was found on the tenth day of fermentation. The optimal cheese whey to date syrup ratio was 50:50. Ascorbic acid decreased the yield of BC but improved the physical properties of the product. The results of the XRD and TGA tests showed that as the concentration of ascorbic acid in the medium increased, the degree of crystallinity and thermostability also increased. Ascorbic acid, regardless of the dose, had no effect on the morphology and diameter of BC fibres.

Some residues, especially in liquid form, for example, potato wastewater (from starch production), have no specific application and cannot be discharged into sewage due to the high COD and BOD values. Until recently, potato wastewater was managed by sprinkling it on meadows and farmland. This solution enriched the soil with nitrogen compounds assimilated by plants, and it was also cheap and easy to implement. However, when there is prolonged irrigation with potato wastewater, the soil becomes clogged and loses water permeability, and this method also leads to water eutrophication (Piwowarek et al. 2022). Ciecholewska-Juśko et al. (2021) used potato wastewater to cultivate *K. xylinus* bacteria (28 °C, 7 days). The yield of BC synthesised from potato wastewater (diluted 1:1 with water) was comparable to the production of BC in the HS medium. BC obtained from potato wastewater did not differ from BC obtained from the HS medium in terms of structure, physical and chemical properties and did not show cytotoxic properties. BC from the side-stream, after soaking with an antiseptic, exerted an antimicrobial effect against bacteria of the species *Staphylococcus aureus* and *Pseudomonas aeruginosa*, just like BC from the HS medium. Ciecholewska-Juśko et al. (2021) found that potato wastewater is a suitable nutrient source for the production of cellulose by *K. xylinus*. The synthesis of BC from this kind of residue makes it possible to valorise this problematic waste material, while reducing the production costs of BC, which enables a wider use of this biopolymer in industry, for example, in biomedicine. Ha et al. (2008) conducted fermentation of residue from beer fermentation broth (WBFB) with *G. hansenii* PJK (KCTC 10505BP) under static conditions at 30 °C (336 h). The researchers used six variants of media: untreated WBFB, autolysed WBFB and hydrolysed WBFB, and each variant was also modified by adding glucose. The highest volumetric efficiency was obtained in the untreated WBFB medium + 1% glucose – 13.95 g/L. In the variant with no added sugar, the production of BC reached 8.46 g/L. In the remaining media, the synthesis of BC was in the range of 2.00–7.37 g/L. Crude distillery effluent, which is characterised by a high COD value and therefore constitutes a serious environmental burden, was used to produce BC with acetic acid bacteria (Gayathri and Srinikethan 2019). The researchers used a bacterial strain of the species *Komagataeibacter saccharivorans* BC1, which after 8 days of cultivation (30 °C, static conditions) produced 1.24 g BC/L with a 23.6% reduction in COD. The diameter of the BC fibres obtained from the tested side-stream ranged from 19 to 195 nm, with an average fibre width of 60 nm. For comparison, the fibres of BC obtained from the standard HS medium, depending on the drying method (hot air oven drying method, vacuum freeze-drying method), had diameters in the following ranges: 14–70 nm (average diameter 30 nm) and 20–180 nm (average diameter 33 nm). The crystallinity of BC from crude distillery effluent reached 80.2%. Gayathri and Srinikethan (2019) showed that the use of distillery wastewater to produce BC would be a beneficial solution for both the environment and industry. The proposed solutions can make the wastewater less toxic, while at the same time producing a biopolymer with a potential application.

The main by-products of the sugar industry are molasses and wastewater. Considering the economic value and nutritional potential of the given residues, they should be considered as substrates for the production of bacterial metabolites. An attempt was made to synthesise BC from sugarcane molasses. Machado et al. (2018) used a bacterial strain of the species *Komagataeibacter rhaeticus*. The cultures were carried out in several variants of media (static conditions, 30 °C, 5 days), differing in the content of glucose and/or sugarcane molasses. The media, apart from the carbon sources mentioned, also contained yeast extract and ethanol. The greatest amount of BC was formed (4.01 g/L) in the medium containing 30 g/L glucose and 20 g/L sugarcane molasses. The authors of the publication showed that sugarcane molasses is an effective substrate in the context of BC synthesis. The tested waste material contains sugars (glucose, fructose, sucrose), amino acids, acids, and vitamins and ash, which guarantee the efficient collection of BC. Machado et al. (2018) calculated that the use of sugarcane molasses can reduce the production costs of BC by about 20%.

Cavka et al. (2013) generated BC from waste fibre sludges using bacteria of the species *G. xylinus* ATCC 23770. These waste materials come from pulp mills and lignocellulosic biorefineries. Fibre sludge is characterised by a high content of cellulose and hemicellulose, and a negligible amount of lignin, which makes it suitable for bioconversion without the thermochemical pre-treatment step. However, enzymatic hydrolysis of this side-stream is necessary in order to obtain a hydrolysate with a high glucose content. Cavka et al. (2013) used two types of fibre sludges: SAFS (from a pulp and paper mill using a sulphate-based process) and SIFS (from a lignocellulosic biorefinery using a sulphite-based process). The investigated residues were subjected to enzymatic treatment. The media, apart from the fibre sludge, also contained yeast extract and tryptone. Cultures were carried out under static conditions for 7 days at 30 °C. Cavka et al. (2013) obtained BC at 11 and 10 g d.w./L for, successively, SAFS and SIFS. The tensile strength of wet BC from the waste materials was about 0.04 MPa, while for the BC obtained from a medium based on pure glucose, it was 0.03 MPA. The crystallinity of the BC from these side-streams was slightly lower compared to the control.

Population growth contributes to the increased demand for clothes, covers, bedding (textiles in general), which results in the production of textile residues. Scientific research shows that these materials can be disposed of using bacteria to obtain BC. Hong et al. (2012) tested cotton-based waste textiles as an environment for the production of BC by *Gluconacetobacter xylinus*. The tested residues were treated with an ionic liquid (1-allyl-3-methylimidazolium chloride) and subjected to enzymatic hydrolysis. The culture medium contained peptone, yeast extract and cotton fabric hydrolysate (glucose after hydrolysis – 17 g/L). Hong et al. (2012) also used a control medium containing the same ingredients as the waste medium, with the difference that they used pure glucose at a concentration of 17 g/L. The used side-stream treated with the ionic liquid showed a 5–7 times higher rate of enzymatic hydrolysis, and also gave a seven-times higher yield of fermentable sugars than the untreated. They obtained 10.8 g of BC/L from the side-stream used, 83% more compared to the control culture. BC from the hydrolysate of the fabric treated with the ionic liquid had 79% higher tensile strength than the BC from the control medium. The pre-treated cotton-based waste textiles can serve as a high-quality carbon source for the production of BC.

Glycerine (crude glycerol) is a by-product of biodiesel production and accounts for 10% by weight of the fuel produced. Glycerine consists of 50–70% glycerol, the remainder being impurities: methanol, free fatty acids, heavy metals and water. In 2016, the annual production of glycerine amounted to 3.8 billion litres (Kieliszek et al. 2020). To be used in industry (pharmaceutical, cosmetic, food, chemical), glycerol must be purified. The glycerine purification process is expensive (deodorisation, bleaching, ion exchange), only large production plants can afford it. The popularisation of biodiesel is hampered by high production costs, which are largely due to the necessity to utilise the side-streams generated. Therefore, much cheaper methods of glycerine biodegradation are needed (Piwowarek et al. 2022). Soemphol et al. (2018) used crude glycerol (from biodiesel production) as a carbon source (30 °C, 14 days) for *Gluconaceter xylinus* BNKC19 which was mixed with the HS broth — achieving a yield of 12.31 g BC/L. In addition, they cultivated the tested strain in a medium containing glycerine and pineapple peel extract (various doses). The most BC was formed in the variant with 50% pineapple peel extract (over 10 g/L).

There are two goals in obtaining BC from side-streams: environmental and economic. From the point of view of environmental protection, the use of industrial by-products for the synthesis of BC will allow for proper waste management, limiting environmental degradation. Moreover, the use of residues for the synthesis of BC will lower the production costs of this biopolymer, which should increase its availability and applicability. Most of the research to date has focused on the efficiency aspects of obtaining cellulose using bacteria and side-streams. The properties of the BC, which determine the applicability of this material, are also of great importance. Water holding capacity, swelling capacity, porosity, the degree of polymerisation, crystallinity, average crystal size, average fibre diameter, mechanical properties and tensile strength — all these parameters depend on various factors, for example, the culture conditions, the type of culture or the composition of the medium (mainly the type of carbon sources). (Urbina et al. 2017). Literature data show that the properties of BC produced from waste materials are very similar and sometimes even better than the properties of this biopolymer obtained from standard, pure culture media (Urbina et al. 2021). On the other hand, it should be remembered that BC made from some residues can become coloured and, moreover, during the synthesis of BC, it can absorb unwanted compounds from the side-streams used. All this means the industrialisation of BC obtained from residues may be difficult, especially in industries characterised by stringent quality requirements (medicine, pharmacy, food industry). Side-streams that guarantee the synthesis of BC with appropriate quality parameters should be pursued. BC should also be subjected to appropriate cleansing treatments to increase its application potential and improve its morphological properties.

## Propionic acid

Propionic acid (PA) is produced by a number of microorganisms (archaea, Gram-positive and Gram-negative bacteria). There are several known routes for the synthesis of PA, depending on the type of microorganism. Overall, the biochemical pathways for PA synthesis are divided into three classes: biosynthetic (citramalate pathway, 3-hydroxypropanoate & 3-hydroxypopanoate/4-hydroxybutanoate pathways — *Haloferax mediterranei*), catabolic (amino acid catabolic pathways) and fermentation (Wood-Werkman cycle — propionic acid bacteria, PAB; acrylate pathway — *Clostridium propionicum*, *Megasphaera elsdenii*; 1,2-propanediol pathway — *Roseburia inulinivorans*, *Salmonella typhimurium*; sodium pumping — *Propionigenum modestum*) (Gonzalez-Garcia et al. 2017). Among the microorganisms mentioned, the best producers of PA, from the industrial point of view, are PAB. Propionic acid bacteria are Gram-positive, non-spore-forming rods 1–5 μm long. They do not show mobility and are classified as anaerobes or relative anaerobes. PAB cells can exist singly, in pairs or in chains. In aerobic conditions, they show pleomorphism (multiformity), they take the shape of the letters Y, V, the so-called “Chinese characters”, or become club-shaped. In the anaerobic environment, they appear as tiny, short sticks (Ahmadi et al. 2017). Due to the environment where they occurr, PAB are divided into two groups: milk (industrial) and skin bacteria (acnes). The main representatives of skin bacteria are *Cutibacterium acnes*, *C. avidum* and *C. granulosum*. Some strains belonging to the skin bacteria group are considered pathogenic microorganisms. In conditions of activated sebaceous glands and reduced immunity of the organism, *C. acnes* bacteria influence the formation of skin lesions (acne vulgaris). The following species are distinguished among milk bacteria: *Propionibacterium freudenreichii*, *Acidipropionibacterium acidipropionici*, *A. jensenii* and *A. theoni* (formerly *P. acidipropionici, P. jensenii*, *P. thoenii*). Industrial PAB are isolated from milk and milk products, cattle rumen, herbivore excrements and sewage. They have also been isolated from rotten fruit juices or granulation lesions in cattle (Piwowarek et al. 2018). PAB is used in the dairy industry (mainly in the production of cheese: Swiss-Emmental cheese, Dutch-Leerdammer, French-Comté, Polish-Tylżycki, Polish-Królewski), in the production of animal silage, and as probiotics in animal nutrition (they are tested for applications as probiotics for humans) (Ahmadi et al. 2017; Piwowarek et al. 2018; Jeantet and Jan 2021).

## Properties and applications of PA

Propionic or propanoic acid is a three-carbon, naturally occurring organic acid (found in apples, strawberries, cheese and human sweat), with its chemical formula being CH_3_CH_2_COOH. The molar mass of PA is 74.08 g/mol and its melting and boiling points are 21 °C and 141 °C, respectively. PA density = 0.99 g/mL. PA is a colourless, oily liquid with a sharp, unpleasant odour (slightly rancid) and a sour and slightly cheesy taste. It dissolves well in water (the addition of salt can separate PA from water, which can be used for acid separation/purification), and it also dissolves in ether, ethanol or chloroform. PA reacts with alcohols and bases to form esters and organic salts. The concentrated form of PA irritates the skin and mucous membranes (Eş et al. 2017).

PA and its salts (calcium, sodium, potassium) have GRAS status; therefore, they can be used in food production. They are used as preservatives, they prevent moulds (packaged sliced bread, rye bread, reduced calorie bread, partially baked bread, rolls, pitta bread, pastries). PA salts are used to preserve animal feed (hay, silage and grain); they inhibit the development of *Aspergillus flavus* and yeast (Coral et al. 2008; Ahmadi et al. 2017). The antimicrobial activity of PA against pathogens was investigated (*Salmonella* spp., *Escherichia coli* O157:H7, *Listeria monocytogenes*) (Mani-Lopez et al. 2012), and can be increased by using PA in combination with other organic acids, such as acetic acid, lactic acid, malic acid and citric acid (Eş et al. 2017). Nearly 80% of the propionate produced is used in the food and feed industry. The remainder is used in the production of: solvents (alkyl propionate esters), anti-inflammatory agents (synthesis of propionic anhydride and chloropropionic acid), herbicides (synthesis of sodium 2,2-dichloropropionate), aromas (a precursor for the chemical synthesis of propionic ether and benzyl propionate, perfume production), plastics, plasticisers, auxiliary substances for textiles and rubber (a precursor for the synthesis of cellulose acetate propionate) (Piwowarek et al. 2018; Ammar and Philippidis 2021) (Fig. 2).

## Industrial PA production

World production of PA is approx. 450 thousand tons/year. The largest producer of PA acid is BASF (Germany), which covers 90% of the world demand for this compound. The remainder is produced by Dow Chemical (USA), Eastman Chemical (USA) and Perstorp (Sweden). On an industrial scale, PA is produced only by chemical means from petrochemical products: the Reppe and Larson processes. In the first one, PA is produced from ethylene (derived from oil refining), carbon monoxide and water vapour, and in the Larson process from ethanol and carbon monoxide in the presence of boron trifluoride (Gonzalez-Garcia et al. 2017; Ammar and Philippidis 2021). PA is produced chemically and not by microorganisms due to the lower production costs and, therefore, lower market price. The price of synthetic PA is approx. USD 1.00/kg, while that of microbial origin is USD 2.00–3.00/kg (Baumann and Westermann 2016; Eş et al. 2017; Ammar and Philippidis 2021). The advantages of the chemical production of PA include the high efficiency and purity of the product obtained (most PA applications require a purity of > 99%, which is achievable in petrochemical production, but very difficult to achieve in fermentation processes). However, it should be remembered that the chemical production of PA is not without its drawbacks: the use of catalysts, toxic reagents and high energy consumption. Due to fluctuations in the price of crude oil, constantly diminishing oil resources, concern for the climate and the environment and the growing popularity of the so-called bio-products, the concept of microbial acid production is gaining more and more popularity (Gonzalez-Garcia et al. 2017). The main limitations of the microbial production of PA are: inhibition of the metabolic activity of bacteria by negative feedback of the end product (PA), which reduces the efficiency of PA production; synthesis of by-products (acetic acid, succinic acid) — these reduce the efficiency of PA synthesis and impede PA purification, which is equivalent to higher costs of the production process; the time-consuming process; the high costs of microbial media, which, like the production of BC, accounts for a significant proportion of the total cost, which increases production costs. The substrates used to create the media, mainly carbon and nitrogen sources, account for more than 30% of the cost of the final product (Yang et al. 2018). In recent years, there have been many reports in which attempts have been made to overcome the above difficulties regarding the microbiological production of PA: genetic and metabolic engineering for PA production, bioreactor cultures/design of bioreactors, immobilisation and culture media consisting of industrial waste.

## PA synthesis

In the context of PA synthesis, bacteria of the species *A. acidipropionici* and *P. fruedenreichii* are most often used, although other PAB species have also been used, for example, *A. jensenii* or *A. thoenii* (Sarmiento-Vásquez et al. al. 2021). The best producers of PA are bacteria of the species *A. acidipropionici*, which results from their greater tolerance to stress related to the acidic environment, due to the higher activity of H^+^-ATPase compared to other PAB. Due to the hydrophobicity of acids (propionic and acetic) and the cell membrane, undissociated acids (in an acidic environment) can diffuse through the bacterial membrane into the cytoplasm and then dissociate; therefore, to maintain a functional gradient inside the cell, the ATP-dependent proton pump (H^+^-ATPase) extrudes protons into the extracellular environment (Guan and Liu 2020).

PAB produce PA through the Wood-Werkman cycle, which begins with the conversion of pyruvate to oxaloacetate as a result of the biotin-dependent methylmalonyl-CoA carboxytransferase. The oxaloacetate is then reduced by malate and fumarate to succinate, which is acetylated by succinyl-CoA synthetase to succinyl-CoA. With the participation of coenzyme B12 and the mutazmethylmalonyl-CoA, succinyl-CoA is transformed into R-methylmalonyl-CoA. This, in turn, in a reaction catalysed by methylmalonyl-CoA epimerase, is isomerised to L-methylmalonyl-CoA, which is converted to propionyl-CoA by the action of methylmalonyl-CoA carboxyltransferase. Transferase-CoA releases CoA, thanks to which propionate is produced (Piwowarek et al. 2018). The key enzymes in the Wood-Werkman cycle are methylmalonyl-CoA carboxytransferases and CoA-transferase. The first of these transfers the carboxyl group from methylmalonyl-CoA to pyruvate with the simultaneous formation of oxaloacetate and propionyl-CoA. Tansferase-CoA transfers (reversibly) CoA from propionyl-CoA to succinate, forming succinyl-CoA and the final product (PA) (Vidra and Németh 2018). The most important cofactors which are involved in the regulation of PA synthesis: ATP/ADP, NADH/NAD^+^ and CoA/AcCoA, biotin and coenzyme B12.

The Wood-Werkman cycle is superior to any other metabolic route in terms of its ability to promote PA synthesis consistent with the metabolic goal of energy maximisation (Gonzalez-Garcia et al. 2017; Ammar and Philippidis 2021). PAB reduce pyruvate to propionate in the Wood-Werkman cyclic process, which is combined with oxidative phosphorylation, producing a higher ATP yield. Pyruvate can be used by PAB both for the synthesis of acetic acid (a by-product of PAB metabolism, the so-called compensating metabolite), during which the reduction of NAD^+^ to NADH takes place, and for the synthesis of propionate NADH is consumed (oxidation of NADH to NAD^+^). In this way, PAB modulate the proportions of pyruvate, which are reduced to propionate or oxidised to acetic acid and CO_2_, which maintains the intracellular redox balance (Wang and Yang 2013). Compared to other bacteria, PAB produce the highest concentration of PA, high productivity and acid production efficiency (Vidra and Németh et al. 2018). The advantage of PAB is also their rich enzyme system, which allows them to use many different carbon sources. In addition, literature data show that they are able to grow in media containing by-products of technological processes, which creates the prospect of obtaining metabolites of these microorganisms with the simultaneous disposal of industrial side-streams (Feng et al. 2011; Yang et al. 2018; Castro et al. 2021; Piwowarek et al. 2022). Another advantage of these microorganisms is the fact that some PAB have GRAS and QPS status, thanks to which both living cells of these microorganisms and their metabolites can be used in food production, which is important in the context of PA or another PAB metabolite (e.g. vitamin B12).

The most commonly used systems for PA synthesis by PAB are batch-type fermentations, fed-batch fermentations and continuous cultures (Ahmadi et al. 2017). The relatively low production of PA, unsatisfactory efficiency and productivity of the fermentation processes prompted scientists to look for solutions that would increase the yield and profitability of microbiological production of PA. Literature data show that the kinetics of PA fermentation can be improved by immobilising microbial cells and using special bioreactor cultures: fibrous-bed bioreactors and packed-bed bioreactors (they enable the immobilisation of bacterial cells) (Suwannakham and Yang 2005; Feng et al. 2011; Zhu et al. 2012; Chen et al. 2013; Wallenius et al. 2015; Yang et al. 2018). Suwannakham and Yang (2005) found that immobilised PAB cells (*A. acidipropionici*) produced significantly greater amounts of PA (71.8 g/L) than free cells (52.2 g/L). Compared to free cells, immobilised cells synthesised: 20–59% more propionate, 17% less acetic acid and 50% less succinate. Similar observations were made by Chen et al. (2013). In studies by Yang et al. (2018), the use of fibrous-bed bioreactors increased both the efficiency and productivity of PA production with soy molasses and CSL compared to the classic stirred tank reactors, in turn: from 0.39 to 0.42 g/g and from 0.35 to 0.81 g/L/h. In the same study, batch cultures were also carried out in flasks, where the carbon source was pure glucose. The productivity of the process was 0.16 g/L/h. Other research teams also made similar observations (Feng et al. 2011; Zhu et al. 2012). Wallenius et al. (2015) used packed-bed bioreactors — the results obtained allowed them to conclude that a given type of bioreactor guarantees stable and faster fermentation, higher productivity, efficiency and a higher concentration of PA.

## Factors influencing PA synthesis

PA biosynthesis by PAB depends on: the temperature, the pH of the medium, the composition of the medium (sources of carbon, nitrogen, vitamins) and the type of culture. PAB are mesophilic microorganisms, but they are resistant to high temperatures, they can survive for up to 20 s at 70 °C (some strains can withstand 76 °C for 10 s). Temperature is an important factor in the efficiency of PAB synthesis of PA. Coral et al. (2008) found that regardless of the carbon sources used (glycerol, sodium lactate, sugarcane molasses), an increase in the culture temperature from 30 to 36 °C resulted in weaker PA synthesis (*A. acidipropionici*) and a poorer cell biomass yield. Piwowarek et al. (2019) obtained a higher yield of PA synthesis (*P. freudenreichii*) at 37 °C–0.32 g/g (30 °C–0.30 g/g). Literature data show that the optimal temperature for PA production is in the range of 30–37 °C, depending on the bacterial strain. The optimal pH for PAB is 6.0–7.0 (Piwowarek et al. 2019). The limit of the minimum–maximum pH values is 4.5–8.0. Lowering the pH below the optimal value reduces the rate and efficiency of PA synthesis, and below 4.0 there is complete inhibition of the metabolic activity of PAB (Coral et al. 2008). This is why it is so important to control pH during fermentation (Piwowarek et al. 2019).

The basic carbon sources for propionic bacteria are saccharides (glucose, lactose, fructose, galactose, sucrose, maltose, raffinose) (Feng et al. 2011; Yang et al. 2018; Ammar and Philippidis 2021), glycerol, erythritol (Falentin et al. 2010; Wang and Yang 2013) and organic acids (lactic acid, gluconic acid) (Coral et al. 2008; Falentin et al. 2010). The maximum theoretical yield of PA synthesis from glucose is 0.55 g/g, while the ratio of PA to acetic acid is 2:1. With glucose as the carbon source, the bacteria produce a compensating metabolite, acetic acid, to maintain the redox balance. The relatively high acetic acid production makes the purification and further processing of PA more complex and costly. A more efficient carbon source is glycerol, the theoretical yield for PA synthesis from this carbon source is 0.80 g/g. The production of PA from glucose, which has a lower degree of reduction (4.00) than PA (4.67), requires the co-production of a more oxidised metabolite (compensatory metabolite), that is, acetic acid (4.00). Glycerol and PA have the same degree of reduction (4.67), thanks to which the conversion of glycerol to pyruvate gives enough NADH for the biosynthesis of PA without the need for acetic acid co-production (Wang and Yang 2013). It is manifested by a higher yield of propionate synthesis and a more favourable P/A ratio. As glycerol has very good properties in the context of PA synthesis, scientists have started to use glycerine as a source of glycerol in order to intensify PA production by PAB (Wang and Yang 2013; Piwowarek et al. 2022). A preferred carbon source in the context of PA synthesis is lactic acid which limits the synthesis of by-products and limits the changes in the pH of the medium during fermentation (Coral et al. 2008).

When it comes to nitrogen, PAB obtain this macroelement from peptides, amino acids, ammonium salts and amines. The type and concentration of nitrogen sources significantly affect the microbial production of PA. Some amino acids, such as arginine and aspartic acid, act as a buffer, stabilising and enhancing the fermentation kinetics by reducing the inhibitory effect of acids on PAB (Fröhlich-Wyder et al. 2002). For the cultivation of PAB, for example, yeast extract, peptone, trypticase soy broth, (NH_4_)_2_SO_4_ were used as nitrogen sources. Side-streams were also used: CSL, potato wastewater or the waste cell biomass of propionic acid bacteria (Feng et al. 2011; Wang et al. 2017; Piwowarek et al. 2019, 2021b, 2022). Feng et al. (2011) for the production of PA used sugar cane molasses (hydrolysate, carbon source) and various sources of nitrogen i.e. peptone/yeast extract and the hydrolysate of waste propionibacterium cells. They used PFB-fed-batch fermentation. In the case of pure nitrogen sources (peptone, yeast extract), 91.89 g PA/L was obtained (254 h of cultivation). Replacing peptone and extract with waste PAB cells yielded 79.81 g PA/L after 302 h of fermentation. Feng et al. (2011) found that waste *Propionibacterium* cells can be used for the ecological and economic production of propionic acid by *P. freudenreichii*. Another waste source of nitrogen is potato wastewater (Piwowarek et al. 2021b). The metabolic activity of *P. freudenreichii* was tested in two different media: (1) a medium containing apple pomace, peptone, yeast extract, biotin and others, (2) and a medium consisting of only pomace and potato wastewater, without any pure laboratory compounds. The highest production of propionic acid (14.54 g/L, 0.44 g/g) was obtained in the medium containing apple pomace and pure supplements. Whereas the PA production in the medium with pomace and potato wastewater reached the level of 12.71 g/L (0.42 g/g). *P. freudenreichii* bacteria showed relatively high metabolic activity in an environment with potato wastewater used instead of peptone and yeast extract (Piwowarek et al. 2021b). Probably propionic acid bacteria are able to grow efficiently and produce metabolites in potato wastewater medium because this side-stream is an abundant source of nitrogen. Includes e.g. arginine and aspartic acid (Kowalczewski et al. 2022) which act as a buffer, enhancing the fermentation kinetics (Piwowarek et al. 2021a, b). In addition, potato wastewater is also a source of vitamins from the group B, e.g. riboflavin, which also play an important role in the metabolism of PAB (Kowalczewski et al. 2022; Piwowarek et al. 2022).

The culture media for PA synthesis by PAB were also supplemented with mineral salts, including: manganese sulphate, magnesium sulphate, cobalt sulphate, zinc chloride, magnesium chloride, dipotassium hydrogen phosphate, potassium dihydrogen phosphate and monosodium phosphate (Kagliwal et al. 2013; Wang et al. 2017). In addition to carbon and nitrogen sources, PAB also need vitamins, especially from group B. All PAB strains require pantothenate (B5) and biotin (B7). As PAB do not have the ability to synthesise these compounds, the medium should be supplemented. Some additionally require thiamine (B1) and p-aminobenzoic acid (B10). The metabolism of PAB also requires cobalamin (B12) and riboflavin (B2). The first of these participates in the Wood-Werkman cycle, while the second is a precursor to the synthesis of DMBI, which is necessary for the synthesis of active vitamin B12 by *P. freudenreichii*. Some strains show the ability to produce these vitamins, but in other cases the medium should be supplemented (Falentin et al. 2010).

Metabolic and genetic engineering techniques were also used to intensify the production of PA. Both *P. freudenreichii* bacteria and *A. acidipropionici* (Wei et al. 2016; Piwowarek et al. 2018) were modified. Metabolic/genetic modifications of PAB are some of the most difficult. This is due to several reasons: (1) the high content of guanine and cytosine in the genome of these microorganisms; (2) the presence of strong restriction-modification systems; (3) the Gram-positive nature of the bacteria, which results in a thick cell wall; (4) a limited number of antibiotics acting on the mentioned microorganisms; (5) the limited availability of molecular tools (Falentin et al. 2010). To overcome these difficulties, for example, undirected evolution through genome shuffling was used. As a result of the conducted research, Luna-Flores et al. (2016) obtained a strain (*A. acidipropionici*) that was characterised by higher PA production (40 g/L), efficiency (0.55 g/g) and productivity (0.84 g/L/h) compared to wild-type strains (respectively: 30 g/L, 0.45 g/g, 0.62 g/L/h). Co-fermentation of various microorganisms was also used to synthesise PA. However, the results obtained were not significantly better than monocultures of PAB (Ahmadi et al. 2017). Despite the attempts made, no fermentation process has been developed so far that would meet all the minimum criteria ensuring the economic viability of microbiological production of PA: concentration 100 g/L, yield 0.60 g/g, productivity 1–2 g/L/h (Tufvesson et al. 2013).

## PA production from side-streams

One of the side-streams used in the production of PA with the use of propionic bacteria was whey (Table 2) — a by-product from the production of cheese and casein. Whey consists of lactose (4.5–5.0% w/v), proteins (0.6–0.8% w/v), lipids (0.4–05% w/v) and mineral salts (Jiang et al. 2015). At present, this residue is mainly used as animal feed or fertiliser. Despite this, it is believed that as much as 180 million tons of whey is not subjected to further use (globally, annually). Utilisation of this material, due to the high BOD and COD parameters, is quite problematic and expensive. In addition, there are costs related to the transport and storage of this by-product. Whey is unstable due to the high content of water and biologically active compounds, hence, the susceptibility of this waste material to the uncontrolled growth of microorganisms. All this means that alternative methods of managing whey are still being pursued, for the benefit of the environment and more economical production (Jiang et al. 2015). Kośmider et al. (2010) cultivated (flask culture, static conditions, 30 °C, 120 h) of *P. freudenreichii* strain in media containing different concentrations of lactose derived from whey (2 and 4%), and the media was supplemented with: casamino acid, casitone, K_3_PO_4_, NaH_2_PO_4_ 2H_2_O, biotin, calcium pantothenate, FeSO_4·_7H_2_O, CoSO_4·_6H_2_O, MnCl_2_·4H_2_O, ZnCl_2_, MgCl_2·_6H_2_O. The greatest amount of PA was formed in the environment with a higher concentration of lactose (22.57 g/L, 0.56 g/g) (2–10.02 g/L, 0.50 g/g). Regardless of the whey dose, the P/A ratio in the post-culture fluid was similar (3.75:1, 3.79:1). The best results of PA fermentation in the medium containing whey (+ yeast extract, trypticase) were obtained by Jiang et al. (2015). In their research, they used a wild strain of *A. acidipropionici* ATCC 4875 and a mutant strain (overexpression of the *otsA* gene). Cultures were carried out in a bioreactor at 32 °C, pH = 6.5. For the wild strain, the maximum PA production was achieved by fed-batch fermentation (125 g/L, productivity: 0.50 g/L/h) with immobilised cells (PEI-Poraver in FBB). The mutant produced 135 g PA/L with a productivity of 0.61 g/L/h (fed-batch fermentation with immobilised cells on PEI-Poraver in FBB); the use of the mutant also produced a higher yield of PA synthesis and lower yields of other metabolites (acetic acid and succinic acid). The above results demonstrate the enormous potential of whey in the context of the industrial production of PA, especially with the use of an appropriate culture technique.

In recent years, many studies have also been carried out on the synthesis of PA from sugarcane molasses — a by-product of sugar production (Coral et al. 2008; Feng et al. 2011). Molasses contains about 50% (by weight) of total sugars (sucrose, glucose and fructose). Feng et al. (2011) used sugarcane molasses and the bacteria *P. freudenreichii* CCTCC M207015. The molasses was subjected to acid hydrolysis, and the medium was supplemented with the addition of peptone, yeast extract, NaCl, MnSO_4_ and KH_2_PO_4_. As a result of the fed-batch fermentation in a plant fibre bed bioreactor (PFB), the tested strain produced 91.89 g PA/L at a productivity of 0.36 g/L/h. In addition to PA, the bacteria also produced acetic acid and succinic acid. PA accounted for 79.60% of all acids. In another experiment, Feng et al. (2011) used a waste nitrogen source in the form of hydrolysed waste propionic bacteria cells (simultaneously with molasses), which resulted in the production of 79.81 g PA/L at a productivity of 0.26 g/L/h. PA accounted for 77.58% of all acids. This study showed that molasses and PAB waste cells can be used for the ecological and economic production of PA by the bacteria of the species *P. freudenreichii*. Yang et al. (2018) used soy molasses (from the production of soy protein concentrate) for the production of PA. Soy molasses mainly contains oligosaccharides from the raffinose family (RFO) and sucrose. The industrial use of soybean molasses is limited by the fact that RFO are difficult to digest for animals (ruminants are the exception) and most industrial microorganisms, including yeasts and lactic acid bacteria (Yang et al. 2018). Yang et al. (2018) cultivated *A. acidipropionici* ACT-1 in media containing either non-hydrolysed or hydrolysed molasses (enzymatic hydrolysis), and all variants contained CSL as a cheap source of nitrogen. Culturing the free cells in the variant with soy molasses without hydrolysis produced 21.9 g PA/L with an average yield of 0.39 g/g and a productivity of 0.35 g/L/h. In the variant with hydrolysed molasses, the fermentation kinetics were as follows: 21.2 g/L, 0.46 g/g and 0.42 g/L/h. In addition, scientists cultivated (culture medium: untreated molasses and CSL) the tested strain using FBB (sequential batch fermentation). *A. acidipropionici* produced more than 70 g PA/L, the production efficiency reached 0.42 g/g and the productivity was 0.81 g/L/h. Yang et al. (2018) showed that *A. acidipropionici* bacteria can be used for the cost-effective synthesis of PA with simultaneous disposal of soy molasses without the need for costly pre-treatment or enzymatic hydrolysis of this waste raw material.

An interesting raw material for the microbiological production of PA is lignocellulosic biomass. The following arguments support the use of this kind of side-stream as a culture media: the abundance of cellulose raw materials, their low cost, high sugar content (including glucose) and the possibility of releasing more fermentable sugars through hydrolysis. The conducted research shows that PAB ferment sugars from both the cellulose and hemicellulose fractions, and that they tolerate inhibitors generated during the pre-treatment of the lignocellulosic biomass — acetic acid, furfural, 5-hydroxymethylfurfural and others (Wang et al. 2017). The disadvantage of lignocellulosic biomass is that it requires pre-treatment (enzymatic/acid hydrolysis), which increases production costs (enzymes, energy). Wang et al. (2017) used hydrolysate corn stover (an industrially important, renewable lignocellulosic resource; source of glucose, xylose and arabinose) to produce PA. The hydrolysate was produced in following way: deacetylation, dilute acid pre-treatment and enzymatic hydrolysis (DDAPH). Bacteria of the species *A. acidipropionici* ATCC 4875 were used as the production strain. The scientists used different concentrations of DDAPH, and supplemented the waste material with various nitrogen sources: (NH_4_)_2_SO_4_, yeast extract, soytone, CSL. As a result of HCD fed-batch fermentations, the tested strain produced 64.7 g PA/L with a yield of 0.50 g/g and a productivity of 0.77 g/L/h. For the synthesis of PA, Wang et al. (2020) used corn stalk hydrolysate (physical hydrolysis, enzymatic hydrolysis) and carried out the culture in an expanded bed adsorption bioreactor. The side-stream was additionally supplemented with: CSL, potassium dihydrogen phosphate and cobalt chloride. The bacterial strain of the species *P. freudenreichii* CICC 10019 produced 91.4 g PA/L, the efficiency of PA synthesis was 0.75 g/g and the productivity was 0.35 g/L/h. The parameters of the fermentation kinetics turned out to be more favourable compared to the control medium (glucose as a carbon source, concentration: 85.4 g/L, yield: 0.66 g/g, productivity: 0.33 g/L/h). Stowers et al. (2014) also used corn waste. They cultivated the strain *A. acidipropionici* in a medium containing corn mash enzymatically treated as source of glucose. The yield of PA synthesis was 0.60 g/g.

Castro et al. (2021) also attempted to produce PA from lignocellulosic biomass in the form of sorghum bagasse (a source of glucose and xylose) — a residue produced during the processing of sweet sorghum. Sweet sorghum is constantly gaining in industrial importance, it grows in various climatic conditions, is resistant to drought and is a source of food and fibre (Ammar et al. 2020). Hence, it can be assumed that the tonnage of side-streams from the processing of sweet sorghum will increase. At the moment, sweet sorghum bagasse (SSB) is used industrially as animal feed, soil fertiliser or combustible fuel to generate energy and heat (Ammar et al. 2020). The SSB used by Castro et al. (2021) was pre-treated (acid hydrolysis) and the medium supplemented with tryptic soy broth, MnSO_4_, K_2_HPO_4_ and yeast extract. They used the *A. acidipropionici* CIP 53164 strain, which produced 35.3 g PA/L, the process efficiency reached 0.61 g/g and the productivity was 1.170 g/L/h (sequential batch fermentation with immobilised cells). Ammar et al. (2020) also cultivated PAB (*P. freudenreichii* DSM 4902) in the medium containing SSB (enzymatic hydrolysis). The media was supplemented with: yeast extract, trypticase soy broth, K2HPO4 and MnSO_4_. Flask cultures of SSB hydrolysate allowed the production of more PA (9.9 g/L), a higher yield of PA synthesis (0.51 g/g) and higher process productivity (0.080 g/L/h) than pure glucose (8.5 g/L, 0.44 g/g, 0.070 g/L/h). The P/A ratio was also better in the SSB medium (2.95:1 vs 2.49:1). This may be due to the fact that the tested side-stream contains ingredients that have buffering properties and enhance the kinetics of PA production (an increase in PAB tolerance of the end product – PA). As a result of the bioreactor culture, the tested strain produced slightly more than 22 g PA/L with an efficiency of 0.45 g/g, while the productivity of the process was 0.168 g/L/h. Ammar et al. (2020) enriched SSB with glycerol, as a result of which the efficiency of PA synthesis increased to 0.59 g/g, the P/A ratio reached 8.42:1. The above literature data show that SSB hydrolysate can be successfully fermented by the bacteria of the species *P. freudenreichii*.

Another lignocellulosic raw material used in the production of PA is cassava bagasse, a residue from starch production. Cassava is an important plant in African countries (Africa share – 61%), Asia and Latin America. Annual production of this raw material is approximately 278 million tons. Cassava is increasingly used as a food source, as well as a raw material for the production of economic goods (Tafesse et al. 2021). This results in increasing production of this plant, and this in turn means the accumulation of more and more cassava bagasse, which is used mainly in animal nutrition. A large part of cassava bagasse is thrown onto meadows or fields, which raises serious environmental concerns (Wang and Yang 2013). Therefore, other innovative methods of managing this waste are sought. Wang and Yang (2013) checked the possibility of effective production of PA in a fibrous-bed bioreactor with the participation of *P. freudenreichii* bacteria with cassava bagasse hydrolysate, crude glycerol (carbon sources) and CSL (nitrogen source). The applied conditions produced PA with efficiency of 0.57 g/g, productivity of 0.25 g/L/h and the P/A ratio was 8.68:1.

An interesting lignocellulose material that is gaining more and more popularity in the world of science is cocoa pod husk (CPH) (Sarmiento-Vásquez et al. 2021). It is estimated that around 19 million tons of cocoa biomass residue is produced annually worldwide, which is considered worthless waste, consisting mainly of cellulose, hemicellulose and lignin (Sarmiento-Vásquez et al. 2021). Sarmiento-Vásquez et al. (2021) used CPH to synthesise PA by *A. jensenii* DSM 20274 bacteria. CPH was subjected to chemical and enzymatic hydrolysis, which produced a material containing glucose at a level of 60.5 g/L. The side-stream was supplemented with glycerol (ratio 1:1 relative to glucose from CPH), nitrogen sources and mineral salts. The maximum production of PA was found after 132 h of the process (10.58 g/L). The highest PA production efficiency was achieved after 108 h of cultivation (0.59 g/g).

Most of the industrial by-products used in the laboratory as the media for the synthesis of PA require supplementation with carbon sources (e.g. pure glycerol), nitrogen sources (e.g. yeast extract, tryptone, peptone), minerals or vitamins (e.g. riboflavin, pantothenic acid, biotin, thiamine, cyanocobalamin) (Zhu et al. 2012; Kagliwal et al. 2013; Wang et al. 2017; Piwowarek et al. 2019; Ammar et al. 2020). This practice increases production costs, which makes it difficult to commercialise microbial PA. Piwowarek et al. (2021a) conducted a bioreactor culture (37 °C, 120 h) of the strain *P. freudenreichii* T82 in a medium containing apple pomace and supplements: yeast extract, peptone, phosphate salts and biotin. The tested strain produced 8.01 g PA/L (yield 0.40 g/g) and 2.29 g acetic acid g/L (yield 0.11 g/g). Economic analysis showed that with the yield obtained, production of 1 kg of PA from apple pomace would cost around USD 3.05/kg — the production would not be profitable. It was necessary to improve the production of PA from apple pomace (increasing the fermentation efficiency, reducing the total production costs). Piwowarek et al. (2021b) made an attempt to increase the profitability of PA synthesis from pomace, using potato wastewater — a replacement for expensive nitrogen and vitamin substrates (yeast extract and peptone). During cultivation (flask cultures, 37 °C, 120 h) in a medium consisting only of the mentioned side-streams, the strain *P. freudenreichii* T82 produced, depending on the concentration of carbon sources (glucose + fructose, from apple pomace): 4%: 12.71 g PA/L with a yield of 0.42 g/g (productivity 0.106 g/L/h, P/A ratio 2.53:1); 2%: 6.39 g/L, 0.39 g/g, 0.053 g/L/h, 2.15:1. To enhance the kinetics of PA fermentation, Piwowrek et al. (2022) enriched the medium containing apple pomace and potato wastewater with glycerine (an efficient source of carbon in the form of glycerol). The *P. freudenreichii* DSM 20271 strain produced 8.15 g PA/L at a yield of 0.48 g/g, the P/A ratio in this cultivation variant was 3.68:1. The results obtained showed that potato wastewater can be a cheap source of nitrogen for PAB during co-fermentation with other residues. In turn, the co-fermentation of sugars and glycerol (from side-streams) increases the efficiency of PA production and reduces the synthesis of acetic acid, which results in a higher P/A ratio. The lower the concentration of by-products in the post-culture fluid, the easier and cheaper the PA purification. The advantage of the presented technology is that the applied waste media did not require any additional supplementation (often with expensive laboratory preparations), and the yields obtained of PA synthesis were comparable with other documented results.

This article shows that in recent years many steps have been taken to industrialise the microbial production of PA — development of bioprocesses (immobilisation, bioreactors design), improvement of strains (metabolic/genetic engineering) and use of industrial side-streams as culture media. After all, biological PA production is still less profitable than chemical synthesis. It seems that to reduce the costs of microbial PA synthesis to a level that could compete with the chemical industry, it is necessary to overcome the defence mechanisms of PAB (strong restriction-modification systems) (Falentin et al. 2010), which limit the interference with their genes. Thanks to which it will be possible to construct better, more efficient strains (increasing the flow of carbon for the synthesis of PA, limiting the synthesis of by-products, increasing tolerance to an acidic environment, reducing end-product inhibition, giving bacteria cellulolytic properties — elimination of the enzymatic hydrolysis stage of lignocellulosic raw materials). Screening of strains characterised by efficient natural PA production should also be carried out — in some parts of the world or in the public perception of GMO, this still raises a number of controversies. The cultivation of improved production strains with the simultaneous reprocessing of residues may contribute to increasing the competitiveness of the microbiological synthesis of PA and improving the quality of the natural environment (waste management, reducing the carbon footprint of side-streams). Further industrial waste materials should also be continually tested, looking for those whose composition would stimulate bacteria to effectively synthesise PA. To increase the profitability of microbiological PA synthesis, it is also possible to use statistical optimisation methods, using several different side-streams at the same time, which would guarantee the availability of appropriate amounts of carbon sources (glycerol, glucose, lactic acid), nitrogen sources (arginine, aspartic acid), vitamins (biotin, niacin, pantothenate, thiamine) or minerals (Piwowarek et al. 2022).

## Vitamin B12

Vitamin B12 (cobalamin) is a water-soluble molecule that is essential in the metabolism of many organisms, including humans. The cobalamin molecule consists of a corrin ring (made of four pyrrole units) with the cobalt (Co) atom in the centre. Besides the four N atoms of the pyrrole units, the central Co ion is linked to two other ligands (lower and upper). The lower ligand is α-ribazole phosphate (the active form of 5,6-dimethylbenzimidazole, DMBI), which determines the therapeutic activity of vitamin B12 in relation to the human body. The presence of another substituent in the lower ligand position, for example, adenine, results in the formation of the so-called pseudovitamin B12 (an inactive analogue). The upper ligand may be occupied by the 5-deoxyadenosyl group (5′-deoxyadenosylcobalamin), -CH (methylcobalamin), -OH (hydroxocobalamin), -CN (cyanocobalamin). The naturally occurring forms of vitamin B12 are 5′-deoxyadenosylcobalamin, methylcobalamin and hydroxocobalamin (found in food products of animal origin) — sensitive to the action of light. Cyanocobalamin is the form produced by industry (vitamin supplements, fortified foods) due to its greater stability (in the body it is converted into active forms of the coenzyme). The type of upper ligand determines the properties of cobalamin (Martens et al. 2002; Deptula et al. 2015). Vitamin B12 acts as a cofactor in producers’ cells. Cobalamin is essential for microorganisms in the reactions of dehydratase, methylase and ammonia lyase (Martens et al. 2002). The regulation of gene expression is also an important role of vitamin B12 in microbial cells. Cobalamin binds to some mRNAs, affecting translation efficiency, and acts as a cofactor for some transcription regulatory proteins (Klug 2014). In PAB, vitamin B12 participates in the conversion of succinyl-CoA to R-methylmalonyl-CoA — the PA synthesis pathway (Piwowarek et al. 2018).

## The importance of vitamin B12 in the human body/industrial use of vitamin B12

Vitamin B12 is synthesised by bacteria and archaea (e.g. *Pseudomonas denitrificans*, *Propionibacterium freudenreichii*, *Bacillus megaterium*, *Salmonella enterica*, *Clostridium* sp., *Strepromyces* sp., *Nocardia* sp., *Thaumarchaeota* sp.) (Calvillo et al. 2022). Naturally, vitamin B12 occurs in foods of animal origin, it comes from the microflora of animals, which produce vitamin B12 or is accumulated in the animal’s body as a result of the consumption of feed enriched with vitamin B12. Vitamin B12 is present in liver (> 23 μg/100 g), crustaceans (52.4 μg/100 g), fish (8.9 μg/100 g) and meat (9.4 μg/100 g). The source of cobalamin can also be eggs (1.3 μg/100 g) and milk (0.4 μg/100 g), but the concentration of vitamin B12 in these types of products is much lower compared to other potential sources of this compound (Sych et al. 2016; Calvillo et al. 2022). The recommended daily intake of vitamin B12 for adults ranges from 2.0 to 3.0 μg (Piwowarek et al. 2018).

Vitamin B12 in the human body acts as a cofactor of methionine synthase (methylcobalamin) and L-methylmalonyl-CoA (5′-deoxyadenosylcobalamin) mutase, acting in the cytoplasm and mitochondria, respectively. Methylcobalamin is crucial in folate metabolism — the conversion of 5-methyltetrahydrofolate to tetrahydrofolate, ensuring the availability of folate derivatives, important for the synthesis of purines and pyrimidines. In addition, methylcobalamin works as a methionine synthase cofactor in the reaction of homocysteine (folin-dependent) methylation that regenerates methionine, enabling the synthesis of S-adenosylmethionine. Consequently, cobalamin provides the methylations necessary for the synthesis of myelin, phospholipids, proteins and neurotransmitters. 5′-Deoxyadenozylocobalamin catalyses the isomerisation of methylmalanyl-CoA to succinyl-CoA, important in the context of the metabolism of amino acids, odd-chain fatty acids, cholesterol and haemoglobin synthesis (Sych et al. 2016; Wolffenbuttel et al. 2019; Calvillo et al. 2022). Cobalamin deficiency results in the accumulation of methomalonic acid or methylammonium acidosis, which impairs the formation of myelin sheaths and cognitive functions (Moore and Warren 2012). Lack of vitamin B12 can cause disorders of neurological functions, DNA synthesis or cause megaloblastic anaemia. The most severe effects of vitamin B12 deficiency include pernicious anaemia, atherosclerosis, heart disease, neurological diseases (limb paralysis, ataxia, lethargy), increased susceptibility of deoxyribonucleic acid to damage and changes in methylation. In developed countries, vitamin B12 deficiency mainly affects certain social groups. One group are people who choose to follow a vegetarian or vegan diet. Cobalamin deficiency also occurs in the elderly, mainly due to lower meat consumption and a high prevalence of food-bound malabsorption. People at risk of cobalamin deficiency also include pregnant women, breastfeeding women, children and people suffering from immune diseases that cause stomach complications that limit vitamin B12 absorption. Vitamin B12 deficiency is common in low-income countries due to low meat consumption (Pawlak et al. 2013; Watanabe et al. 2013). The bioavailability of vitamin B12 from food is only approx. 50%; it should also be remembered that during food processing (exposure to light, heat treatment) part of the vitamin is degraded or transformed into inactive forms. All this makes the daily intake of cobalamin insufficient, especially in people who do not eat animal products or have problems with intestinal absorption. In all of the above cases, a higher than normal daily intake of vitamin B12 is recommended, mainly through supplementation.

Vitamin B12 is used in the food industry to fortify a variety of nutritional products. Cyanocobalamin is most often used (due to its greater stability during processing, food production). In the feed industry, vitamin B12 is added to fodder for poultry, pigs or calves, most often in doses of 10–30 mg/t. Due to the increasing popularity of vegetarian and vegan diets, supplementation with vitamin B12 is becoming more and more popular. Cyanocobalamin is also mainly used in the supplement industry (mainly due to its price and stability). Vitamin B12 is also widely used in the pharmacological sector where cyanocobalamin and other forms of vitamin B12 are used, namely hydroxocobalamin, methylcobalamin and 5′-deoxyadenosylcobalamin (due to their higher absorption and more sustained serum levels). In the pharmaceutical industry, vitamin B12 is produced in the form of nasal sprays, tablets or injections — it is used in the treatment of pernicious anaemia, vitamin B12 deficiency and cyanide poisoning (Obeid et al. 2008; Watanabe et al. 2013; Calvillo et al. 2022) (Fig. 2).

## Industrial vitamin B12 production

Vitamin B12 for industrial purposes is obtained microbiologically with the use of two species of bacteria: *P. denitrificans* and *P. freudenreichii*. There is also a known chemical way of obtaining cobalamin, including about 70 steps; however, it is too complicated and expensive. Bacteria used in the industrial production of cobalamin are selected for productivity, genetic stability and rapid growth rate. In the industrial production of cobalamin, random mutagenesis or genetic engineering techniques are commonly used to obtain strains that produce high yields of vitamin B12. In this case, *Pseudomonas* bacteria are preferred. Most of the production is based on *Pseudomonas* bacteria, which satisfies approx. 80% of the global demand for this compound (Martens et al. 2002). In Europe, Sanofi is a leader in the production of vitamin B12. The world leader in producing vitamin B12 is China: North China Pharmaceutical Company, Henan Luyuan Pharmaceutical Company, Hebei Yuxing Bio-Engineering Company and CSPC Huarong Pharmaceutical Company. The Chinese patent for the synthesis of cobalamin with the participation of *Pseudomonas* bacteria declares the volume production of this compound to be 281 mg/L (Zhendong et al. 2020; Calvillo et al. 2022). In 2005, the production of vitamin B12 reached 10 tons (market value: approx. EUR 77 million). In 2020, China alone produced 31.40 million tons of cobalamin with a market value of USD 339.48 million. There are reports that predict that in 2027 the global market for vitamin B12 will reach USD 410 million (Calvillo et al. 2022). The continuous increase in the production of vitamin B12 is due to the increasing demand for this compound, which is related to the ageing population, the growing popularity of vegan and vegetarian diets or — in some places in the world — the shortage of meat foods. Both species are characterised by a relatively high efficiency of cobalamin synthesis, while the advantages of *P. denitrificans* bacteria over *P. freudenreichii* are faster growth, shorter production time, lower nutritional requirements, higher volumetric productivity and easier genetic manipulation (Kang et al. 2012). The advantage of *Pseudomonas* bacteria is also the fact that they produce cobalamin under aerobic conditions, which greatly facilitates the production process — no need to use anerostats, easier control of the process. On the other hand, *P. denitridicans* does not have GRAS status and therefore cannot be used in food production. In this case, the species *P. freudenreichii* is the leader, which can be used to enrich food (e.g. for vegans) with vitamin B12 by in situ fermentation.

Vitamin B12 recovery after the fermentation process is based on extraction and purification. The culture fluid or the aqueous suspension of the bacterial cells is exposed to high temperature (80–120 °C for 10–30 min at pH 6.5–8.5). Cobalamin needs to be transformed into a more stable form — cyanocobalamin. For this, the addition of cyanide is used. The cyanocobalamin solution is then clarified by filtration (microfiltration/nanofiltration) and adsorption (XAD resin). The described method is sufficient if vitamin B12 is intended for the fodder industry, when it is precipitated with organic solvents, for example, acetone. If higher product purity is required, for example, in pharmaceutical applications, further adsorption steps (IRA, Alumina resins) are needed. Purified vitamin B12 can be modified to increase its stability or bioavailability e.g. microencapsulation (Nielsen et al. 2012; Sych et al. 2016; Calvillo et al. 2022).

## Vitamin B12 synthesis

There are two known routes for the synthesis of vitamin B12 — aerobic and anaerobic. The first of these occurs, among others, in bacteria of the genus *Pseudomonas*, and involves genes with the *cob* prefix. Genes with the cbi prefix are involved in the synthesis of cobalamin under anaerobic conditions. To effectively produce vitamin B12, bacteria of the genus *Propionibacterium* require both anaerobic and aerobic conditions (in appropriate time intervals). The genome of *Propionibacterium* is equipped with genes with the prefixes *cob* and *cbi* (Falentin et al. 2010; Piwowarek et al. 2020). Cobalamin synthesis by *Pseudomonas* occurs simultaneously with bacterial growth; in the case of *P. freudenreichii* the greatest accumulation of this metabolite takes place in the stationary phase. Both these routes can be divided into four stages: synthesis of ALA (δ-aminolevulinic acid), synthesis of the corrin ring component, construction of the lower axial ligand and the synthesis of cobalamin (Kang et al. 2012). Vitamin B12 biosynthesis begins with the C-5 glutamate backbone. In the first step, the glutamate bound to the tRNA is reduced to glutamate-1-semialdehyde by glutamyl-tRNA reductase. The resulting glutamate 1-semialdehyde is converted by glutamate semialdehyde aminotransferase to 5-aminolevulinic acid (ALA) (intramolecular amino group shift between C-2 and C-1 semialdehyde). Two molecules of 5-aminolevulinic acid condense to form the first pyrrole derivative, the porphobilinogen. The next stage is the polymerisation of four PBG molecules, which results in the formation of a preuroporphyrinogen, which undergoes inversion and cyclisation reactions — the synthesis of biologically active uroporphyrinogen III (a precursor to the cortical system) takes place. Three enzymes participate in the above transformations: 5-aminolevulinic acid dehydratase (hemB), porphobilinogen deaminase (hemC) and uroporphyrinogen synthetase III (hemD). As a result of the action of uroporphyrinogen III methyltransferase, the precursor of the cortical system undergoes metisation at the C-2 and C-7 positions — a prototype of the cortical ring is created. From this point, depending on the pathway, cobalamin synthesis proceeds differently. The anaerobic pathway, after the formation of the corrin ring, begins with inclusion of the cobalt, while in the oxygen-dependent pathway, inclusion of cobalt into the vitamin structure occurs after nine consecutive reactions. Both pathways involve other chelatases. In the aerobic pathway, cobalt chelase is activated with the participation of ATP-derived energy, and in anaerobic conditions, ATP-independent chelatase is used. Another difference in the synthesis of vitamin B12 by the aerobic and anaerobic routes is the contraction of the corrin ring associated with the removal of C-20 carbon. Under aerobic conditions, the C-20 carbon atom is oxidised by the action of molecular oxygen, and, as a result, is excreted as acetic acid. Under anaerobic conditions, the narrowing of the ring takes place due to the presence of the cobalt ion, which can assume various valence states — it participates in the oxidation process, which results in the removal of the C-20 carbon atom in the form of acetaldehyde. The counter-circulation of the corrin ring serves to stabilise the cobalt chelation. Then cobalt adenylation takes place (the upper ligand is formed). The formation of the nucleotide under the macrocyclic ring involves several reactions. First, the aminopropanol arm is attached to the corrin ring followed by its phosphorylation; alternatively, phosphorylation may take place prior to attachment. Then, the phosphor group is activated by adding adenosine-GDP at the expense of one GTP. At the same time, α-ribazole is activated and a transferase reaction takes place replacing adenosine-GDP with α-ribazole, which leads to the formation of coenzyme B12. The complete pathway for cobalamin synthesis consists of approximately 30 genes. The most important are the last stages of the vitamin production pathway, essential for the production of active vitamin B12. They consist of: synthesis, activation and attachment of a lower ligand. In aerobic and aerotolerant microorganisms, DMBI is synthesised in an oxygen-dependent manner from a reduced flavin mononucleotide (an organic chemical compound, an ester of phosphoric acid and riboflavin) under the influence of the BluB enzyme (Martens et al. 2002; Deptula et al. 2015; Piwowarek et al. 2018; Calvillo et al. 2022). In strictly anaerobic microorganisms (*Eubacterium limosum*, *Acetobacterium woodii*), DMBI synthesis is attributed to the *bzaABCDE* gene cluster (*bzaA-bzaB-cobT-bzaC-bzaD-bzaE*) (Hazra et al. 2015; Calvillo et al. 2022). DMBI is then activated by the enzyme CobT (anaerobes) (CobU in aerobes) to α-ribazolophosphate, which is then attached to cobalamin to form the complete active vitamin B12 molecule.

## Factors influencing vitamin B12 synthesis

Cultivation of *Pseudomonas* bacteria for cobalamin production is carried out with aeration and agitation for 3–7 days at 30 °C at a medium pH of approx. 7.0. The efficiency of vitamin B12 synthesis depends on the dissolved oxygen concentration (DOC) as well as the level of the inlet CO_2_ fraction. The increase of bacterial biomass and the production of vitamin B12 depend on oxygen, the production of biomass is favoured by a higher DOC, and the synthesis of cobalamin by a lower one. Li et al. (2012) attempted to validate the multi-step DOC control strategy in a large volume of culture – 120,000 L fermenter. The concentration of dissolved oxygen was changed from 8–10% (20–48 h) to 2–5% (49–106 h) and to 2% (107–168 h) by gradually reducing the aeration and mixing rates — 198.80 mg cobalamin/L was obtained, 20% more compared to the one-step DOC control strategy. Li et al. (2008) showed that supplementation of the culture medium with Zn^2+^ has a positive effect on the synthesis of ALA and PBG (precursors of vitamin B12). Co^2+^ and DMBI also have a beneficial effect on the production of vitamin B12. Optimisation of the initial Zn^2+^, Co^2+^ and DMBI concentrations increased the cobalamin synthesis by *P. denitrificans* by 13% — from 69.36 to 78.23 μg/mL. Literature data show that the addition of betaine also has a positive effect on the synthesis of vitamin B12. Betaine acts as a methyl donor in the biosynthesis of vitamin B12, enhancing the synthesis of important intermediates: ALA, glutamate, glycine and methionine (Li et al. 2008; Xia et al. 2015a, 2015b). Too high a concentration of betaine in the breeding medium can inhibit the growth of *P. denitrificans* bacterial cells; therefore, an effective betaine dosing strategy was developed — to counterbalance the negative effect of betaine on cell growth and the positive effect on cobalamin production. This increased the synthesis of cobalamin by approx. 10% compared to the control sample (Li et al. 2008). Cheng et al. (2014) proved that the synthesis of cobalamin can be improved by adding rotenone to the medium; it is an inhibitor of the respiratory chain (it disrupts oxygen consumption). It was found that the addition of an inhibitor at a dose of 5 mg/L enhances the glycolytic flow in the metabolism of *P. denitrificans* bacteria by activating key glycolytic enzymes (phospho-frutokinase and pyruvate kinase), which accelerates the synthesis of glutamate and 5-aminolevulinic acid (crucial in the synthesis of vitamin B12). Contrary to the bacteria *P. freudenreichii*, genetic modifications of the *P. denitrificans* strain resulted in a significant improvement in the efficiency of cobalamin synthesis. The strain used by Sanofi is the result of random mutagenesis and molecular biology techniques. The improved strain was derived from a strain known as MB-580, in which the French company Rhône-Poulenc amplified several *cob* genes involved in vitamin B12 biosynthesis, resulting in a high-yielding strain producing 200 to 300 mg cobalamin/L (Martens et al. 2002).

*Propionibacterium* bacteria produce vitamin B12 with high efficiency only at a very low concentration of oxygen; however, for the synthesis of active cobalamin, they nevertheless require its presence in the culture medium — for the synthesis and attachment of DMBI. Therefore, the production of vitamin B12 using strains of *Propionibacterium* is divided into two stages. In the first stage, which lasts 3 days, the bacteria are cultivated under anaerobic conditions, thanks to which the bacteria grow and synthesise the vitamin B12 intermediate — devoid of the DMBI group (which determines the activity). The culture is then gently aerated for 1–3 days to produce DMBI (from riboflavin) and attach it to the vitamin B12 molecule (Martens et al. 2002; Deptula et al. 2015; Sych et al. 2016; Calvillo et al. 2022). Although *P. freudenreichii* bacterial strains are capable of synthesising DMBI, the production of this compound by the bacteria is low. If the availability of DMBI is limited, bacteria accumulate the incomplete forms (cobinamide or pseudovitamin B12) instead of active vitamin B12. In order to improve the efficiency of active cobalamin synthesis, the culture media was supplemented with DMBI or its precursors (riboflavin, nicotamide) (Chamlagain et al. 2016). More efficient production of cobalamin can also be obtained by adding ALA and/or Co^2+^ to the medium (Martens et al. 2002; Hajfarajollah et al. 2015). In addition, the efficiency of cobalamin synthesis can be increased by optimising the composition of the fermentation media by selecting the appropriate types and amounts of: carbon and nitrogen sources, mineral salts and betaine (Kośmider et al. 2012; Haifarajollah et al. 2015; Hedayati et al. 2020). *P. freudenreichii* bacteria are cultivated for cobalamin production at a temperature of 30 °C and a pH of 6.5–7.0. During the production of cobalamin, the acids formed as a result of fermentation need to be neutralised, especially PA, which in high concentrations rapidly lowers the pH of the medium, becomes toxic and limits cell growth (negative feedback). One way is to regularly dose with alkalising substances — NaOH, ammonia water (Piwowarek et al. 2022). Wang et al. (2012) conducted a batch fermentation of *P. freudenreichii* using the EBA (expanded bed adsorption bioreactor) system with high biocompatibility resin (ZGA330) — an in situ product removal (ISPR) technique. PA was recovered by semi-continuous recirculation of the unfiltered broth, which eliminated the negative feedback inhibition of PA. The production of vitamin B12 increased by approx. 18%, from 36.40 to 43.04 g/L. To increase the production of cobalamin by P*. freudenreichii*, genetic methods were also attempted (Piao et al. 2004a, b). However, due to the fact that the metabolic/genetic modifications of PAB are one of the most difficult (Falentin et al. 2010) and due to the complexity of the vitamin B12 synthesis pathway, the final results of genetic manipulations were not sufficiently satisfactory. Therefore, it seems that the most effective methods of improving the synthesis of cobalamin by *P. freudenriechii* are related to optimisation of the fermentation process and the culture medium.

## Vitamin B12 production from side-streams

Synthetic culture media make the industrial production of vitamin B12 an expensive process. Another problem is the extraction of cobalamin from the culture medium. It is expensive due to the low concentration of the vitamin. A possible way to reduce production costs is the use of cheap materials, for example, in the form of food waste, agroindustial and industrial side-streams (e.g. cooking oils, molasses, fruit pomace, glycerine) (Table 3).

Tofu is a traditional oriental food made from soybeans which is an abundant source of protein, and, therefore, it is gaining more and more popularity all over the world, especially among vegans. The production of tofu is based on a multi-stage process: raw soybean selection, soaking, milling, heating soy milk, filtration, addition of coagulants, pressing, packing (Zheng et al. 2020). To produce 80 kg of tofu, 60 kg of soybeans and 2700 kg of water are needed. The technological process produces 70 kg of solid side-streams and 2610 kg of sewage (Zheng et al. 2020). Solid waste is not a major environmental problem, it is disposed of — it is used as fodder or bio-fertiliser. The greatest threat is tofu wastewater, due to its high tonnage and high COD and BOD parameters (17,000–26,000 mg/L, 5800–7900 mg/L). Liquid waste is usually illegally discharged into water reservoirs. It is therefore necessary to find a way to valorise this waste (Yu et al. 2015). Yu et al. (2015), to produce vitamin B12, cultivated the strain *P. freudenreichii* DSM 20270 in tofu wastewater. They investigated the effect of different wavelengths of light on the growth rate of bacteria and on cobalamin synthesis. They used red, green and blue light emitting diodes (LEDs), with dark conditions used as the control. The results obtained showed that the tested strain is able to produce vitamin B12 in the tofu wastewater. Most of this compound was formed under blue light conditions (10 μg/mL). The results of the transcription of the *cbiB* gene (involved in the vitamin B12 synthesis pathway) showed that blue light induced the synthesis of this enzyme, which resulted in more efficient production of cobalamin.

In the 2020/2021 season, the global consumption of vegetable oil amounted to over 200 million tons. Waste cooking oil (WCO) is produced by frying raw materials of plant or animal origin with the use of edible vegetable oils (sunflower oil, palm oil, rapeseed oil) — in the food industry, in households, restaurants, hotels and other catering outlets. Consumption of WCO negatively affects human health. During the frying process (160–200 °C), vegetable oils (consisting of triacylglycerols, TAG) undergo many physical and chemical modifications. As a result of the oxidation, hydrolysis and polymerisation of TAG compounds, compounds with mutagenic, carcinogenic, neurotoxic and hepatotoxic properties are formed. A large amount of WCO generated also harms the environment. WCO most often goes into sewage systems or water reservoirs, where it generates unpleasant odours, blocks outflows (drains) and pollutes land and water habitats. WCO has compounds that accumulate in the environment for many years: increasing the organic load on water sources, creating a thin layer above the water surface that reduces the concentration of dissolved oxygen in the water (required for underwater species) (Awogbemi et al. 2021). There is no doubt that new methods of managing this waste are needed. One option is the production of vitamin B12 with *P. freudenreichii*. Haifarajollah et al. (2015) investigated the ability of *P. freudenreichii* PTCC 1674 to produce cobalamin in a medium containing waste frying sunflower oil (WFO). The synthesis of this compound was assessed depending on the concentration of WFO. The medium containing 4% WFO was characterised by the highest production of vitamin B12. Then, using two optimisation steps (Placket-Burman and RSM), the concentrations of some components of the culture medium were optimised. The research results showed that DMBI, cobalt chloride, iron sulphate and calcium chloride were of the greatest importance in the context of cobalamin synthesis. With the help of the RSM tool, optimised concentrations of the indicated nutrients were determined, which produced vitamin B12 at 2.74 mg/L. Research by Haifarajollah et al. (2015) provided valuable information on a viable carbon source for industrial cobalamin production.

Rice bran oil (Hedayati et al. 2020) has been used to produce vitamin B12 with the participation of PAB. Rice bran is a by-product of rice processing — the second largest grain crop worldwide. The global production of rice, each year, is approx. 650 million tons. As a result of the milling of the raw material, 29 million tons of rice bran are produced annually (Sohail et al. 2017). This is rich in carbohydrates, lipids, protein and dietary fibre, and contains significant amounts of mineral elements: including phosphorus, potassium, magnesium, calcium and manganese (Hedayati et al. 2020). The waste is mainly used as animal feed, but rice bran is also a potential source of oil, which, in addition to food values, can also be used as a component of culture media. It is a rich source of fatty acids (linoleic, oleic, palmitic acids), which are a potential source of carbon for microorganisms (Hedayati et al. 2020). Hedayati et al. (2020) used the strain *P. freudenreichii* PTCC 1674, with the culture was carried out in two stages: anaerobic conditions were used for the first 3 days, and aerobic conditions for the next 3 days. The culture medium, in addition to rice bran oil, contained: peptone casein, yeast extract, L-glutamic acid, betaine hydrochloride, (NH_4_)_2_HPO_4_, FeSO_4_·7H_2_O, ZnCl_2_, MgCl_2_·6H_2_O, DMBI, CaCl_2_·2H_2_O and CoCl_2_·6H_2_O. Optimisation (temperature, concentration of rice bran oil, DMBI, elemental solution) of the vitamin B12 synthesis process by the tested strain achieved production of this metabolite at 2.94 mg/L. It has been found that *P. freudenreichii* PTCC1674 can grow and produce cobalamin from rice bran oil as a new carbon source.

For the production of vitamin B12, Wang et al. (2020) used the bacteria *P. freudenreichii* CICC 10019 and corn stalk (lignocellulose waste). The residue was supplemented with the additives: CSL, potassium dihydrogen phosphate and cobalt chloride. Wang et al. (2020) used fed-batch fermentation with hydrolysed corn stalk in an expanded bed adsorption bioreactor (EBAB). Cultures were carried out at 30 °C and pH control was automatic (the pH was maintained at 7.0 using 12% ammonia solution). After 256 h, the tested strain produced 47.6 mg/L of vitamin B12.

Another side-stream tested for the possibility of producing vitamin B12 with the participation of *P. freudenreichii* was glycerine. Kośmider et al. (2012) found that crude glycerol can be a carbon source for *P. freudenreichii* in the context of vitamin B12 synthesis. The authors of the publication used media which, apart from glycerine, contained different amounts of supplements (depending on the variant of the medium): biotin, Ca pantothenate, CoSO_4_·6H_2_O, NaH_2_PO_4_·2H_2_O, DMBI and casein hydrolysate. The maximum production of vitamin B12 in the optimised medium was 4.01 mg/L.

The possibility of obtaining vitamin B12 from the liquid acid protein residue of soybean (LAPRS) (Acosta de Asis et al. 2019), which is obtained as an effluent during the soybean-isolated protein process (SIP), was also investigated. A single SIP plant generates approximately 50,000 m^3^ of LAPRS per month. LAPRS consists mainly of carbohydrates and proteins, and also contains a number of micro- and macro-elements (Acosta de Asis et al. 2019). This waste is distinguished by a high biochemical oxygen demand (> 20,000 mg O_2_/L), so it cannot be disposed directly into the environment — it requires expensive treatment, which of course involves economic losses. Accordingly, Acosta de Asis et al. (2019) attempted to valorise LARPS by using this side-stream as a medium for *P. freudenreichii* ATCC 13673 for the synthesis of vitamin B12. Cultures were carried out at a temperature of 30 °C, for the first 72 h under anaerobic conditions, and then for 96 h under microaerophilic conditions. Optimisation of cobalamin production (selection of appropriate concentrations of individual supplements: potassium phosphate, cysteine chloride, iron sulphate, cobalt chloride and DMBI) with the participation of the tested strain produced vitamin B12 at 0.6 mg/g of bacterial cells.

From a food production point of view, the addition of cobalt and/or DMBI to the production environment is not allowed. Supplementation of media with these ingredients in order to obtain cobalamin for pharmaceutical purposes, due to the high prices of these reagents, is associated with large financial outlays, which of course reduces the profitability of the process. Therefore, side-streams that would guarantee efficient production of vitamin B12 without additional supplementation should be pursued. Piwowarek et al. (2022) investigated three residues: apple pomace, waste glycerine and potato wastewater. Cultures were carried out for 5 days at 30 °C in media containing various proportions of the above-mentioned raw materials, without any supplementation (stationary conditions). Bacteria synthesised the highest amount of vitamin in the medium consisting of apple pomace and potato wastewater (289.80 µg/100 g of wet bacterial biomass). Waste glycerine was of the least importance for the production of vitamin B12. Piwowarek et al. (2022) suspect that apple pomace and potato wastewater may be a source of the precursors of vitamin B12 (cobalt and riboflavin). Hence, the more efficient production of this metabolite in media in which both these side-streams were the dominant components.

In addition to *P. freudenreichii*, bacteria of the species *Pseudomonas denitrificans* were also used for the disposal of side-streams with the simultaneous production of vitamin B12. Li et al. (2013) attempted the microbiological synthesis of vitamin B12 from beet molasses. The culture medium consisted of molasses, sucrose, betaine, (NH_4_)_2_SO_4_, MgSO_4_·7H_2_O, ZnSO_4_·7H_2_O, CoCl_2_·6H_2_O and DMBI. As a result, 181.75 mg of vitamin B12/L was obtained. Beet molasses contains compounds that favour the production of cobalamin by *P. denitrificans* — sucrose, glutamate and betaine (Li et al. 2008, 2013). The disadvantage of using beet molasses is that it must be pre-treated (acidification, heat treatment). In addition, some components of beet molasses may negatively affect the viability of the bacteria or the stability of fermentation — colouring substances, heavy metals (Xia et al. 2015b). Therefore, an attempt was made to produce vitamin B12 from other side-streams less toxic to the tested bacteria —maltose syrup (a cheap product obtained from corn starch production as a result of enzymatic or acid hydrolysis) and CSL (Xia et al. 2015b). The results obtained showed that maltose syrup and CSL can be used as substrates for the effective production of cobalamin by *P. denitrificans* (medium: maltose syrup from corn starch, CSL, betaine, (NH_4_)_2_SO_4_, MgSO_4_, KH_2_PO_4_, ZnSO_4_·7H_2_O, CoCl_2_·6H_2_O and DMBI). In the optimised medium containing the residues, the bacteria produced 198.27 mg cobalamin/L. The result obtained was similar to the culture in which the carbon source was refined sucrose (198.80 mg/L) and higher compared to the beet molasses medium (181.75 mg/L) (Li et al. 2013; Xia et al. 2015b).

Vitamin B12 deficiency, until recently considered extremely rare, except in strict vegans, is now quite common — also among people following vegetarian diets (e.g. lacto-vegetarianism). Vitamin B12 deficiency may also result from intestinal malabsorption, ileal disease, lack of transcobalamin I or II (a transport plasma protein) or an excessive increase in the intestinal microflora that uses vitamin B12 for its own purposes. Therefore, vitamin B12 deficiency appears to be a global problem. Taking into account the growing social awareness related to concern for the environment or animal welfare, and thus the changing food trends (growing popularity of vegetarian diet), it can be assumed that in the coming decades the demand for vitamin B12 supplementation or plant products enriched with this compound will increase. There will be a fundamental shift in consumption patterns from animal protein to vegetable protein in the coming years. This is a huge challenge for the world of science, because, regardless of the food source, the nutritional value of the consumed products must be preserved, and the most important nutrient that cannot be guaranteed by any plant in the diet is vitamin B12. *P. freudenreichii* is the only microorganism with GRAS status (Piwowarek et al. 2018) that has the ability to synthesise the active form of vitamin B12, which makes it a unique candidate for the production of food, for example, of plant origin (Xie et al. 2018, 2019, 2021), enriched in cobalamin by in situ fermentation.

There are reports in the literature examining the possibility of producing vitamin B12 using cereal bran (a by-product of the milling process of cereals), for example, wheat bran. Cereal bran contains dietary fibre, proteins, vitamins, polyphenols and phytosterols. Most of the bran is used as animal feed. Due to the negative influence of bran on the technological and organoleptic quality of products made from it, it is not widely used in the food industry. Refined white flour is preferred by most consumers. The texture and taste properties of products made with bran are perceived as less attractive compared to products made with refined flour. For example, in the production of bread, supplementation with bran usually weakens the structure and baking quality of the dough, reduces the volume of bread and the elasticity of the crumb and lowering the overall product quality. The addition of bran to bakery products is only successful when processing techniques are used, for example, pre-fermentation. Hence, the need to valorise this waste. One of the options is the in situ fermentation of this type of raw material to fortify vitamin B12. Fermented bran could be used, for example, in the production of bread or extruded products (Xie et al. 2018, 2019, 2021). Xie et al. (2018) fermented wheat bran using *P. freudenreichii* DSM 20271 bacteria. After 7 days of cultivation, the material was found to contain vitamin B12 at a level of 155 ng/g of dry wheat bran. In a subsequent study, Xie et al. (2019) conducted wheat bran co-fermentation with *P. freudenreichii* DSM 20271 and *Levilactobacillus brevis* ATCC 14869. The lactic acid bacteria strain was used to improve the microbiological safety of fermented bran (the bran was not sterilised) and to improve the microbiological stability and sensory properties of the material. After 3 days of fermentation, the production of vitamin B12 reached 332 ng/g dry substance of the product. The presented results show that *P. freudenreichii* bacteria can produce vitamin B12 in the wheat bran. The same research team (Xie et al. 2021) checked the possibility of supplementing other cereal brans with vitamin B12 — rye bran, oat bran, rice bran and buckwheat bran. They used the co-fermentation of the same bacterial strains (*P. freudenreichii* DSM 20271 and *L. brevis* ATCC 14869). Bacteria synthesised cobalamin in each of the tested materials, with the most vitamin B12 formed in rice bran — 742 ng/g rice bran dry substance. The co-fermentation of *P. freudenreichii* and other species presents a promising opportunity to enrich plant materials with vitamin B12, which could then be used in the production of a wide variety of foods. Vitamin B12 yields in these studies are lower compared to other media, but, in the context of food fortification, the results presented show that there is a possibility of fortifying vitamin B12 in food (e.g. vegetables), which may facilitate the consumption of the daily recommended dose of this cofactor, especially for vegans — without the need to supplement the diet with pharmaceutical products.

## Polyhydroxyalkanoates/polyhydroxybutyrate

Polyhydroxyalkanoates (PHAs) are other bacterial metabolites with high industrial potential. They are natural chemical compounds (of biological origin, in the form of polyesters) that are biodegradable (100%) (Mangaraj et al. 2019). PHAs are a backup material for bacteria (carbon and energy reserves) located in the cytoplasm in the form of inclusion bodies surrounded by a protein-lipid membrane, called granules (Wältermann et al. 2005). Bacteria can accumulate PHAs at up to 80–90% of their dry cell weight. If necessary, the PHAs are degraded and can therefore be used by microorganisms as a source of carbon and energy. PHAS are synthesised by various groups of microorganisms. Some bacteria (e.g. *A. eutrophus*, *Protomonas extorquens*, *Protomonas oleovorans*) accumulate PHAs with an excess of carbon, accompanied by an incorrect pH or a deficiency of a specific nutrient, for example, sulphur, phosphorus, nitrogen, oxygen, magnesium. There are examples of bacteria that do not require any food restrictions for the synthesis of PHAs (production depends on the growth of bacteria): bacteria *Alcaligenes latus*, a mutant strain of *Azotobacter vinelandii* or a recombinant strain of *E. coli* bacteria (Monroy and Buitrón 2020; Brojanigo et al. 2022; Tyagi et al. 2022). The bacteria for the production of PHAs are mainly obtained from contaminated land, food waste materials and sediments (Kingsly et al. 2022).

## Properties and applications of PHAs

So far, approx. 300 species of microorganisms (mainly bacteria, both Gram-positive and Gram-negative) capable of synthesising PHAs and approx. 150 different structures of this polyester have been identified (Kourmentza et al. 2017). Among the bacteria capable of accumulating large amounts of PHAs and their copolymers are wild and recombinant strains of the following bacteria: *Cupriavidus necator* (formerly *Ralstonia eutropha*), *Azotobacter* sp., *Bacillus* sp., *Pseudomonas* sp., *Burkholderia* sp., *Cyanobacteria*, *Chromobacterium* sp., *Methylobacterium* sp., *Thermus thermophilus*, *E. coli* (Anjum et al. 2016). PHAs are divided into two groups (by the number of carbon atoms in the chain of branched polymers and the type of homopolymers or heteropolymers producing monomeric units): short-chain (scl-PHAs) and medium-chain (mcl-PHAs). Examples of scl-PHAs are poly(3-hydroxybutyrate) [P(3HB)], poly(4-hydroxybutyrate) [P(4HB)], poly(3-hydroxyvalerate) [P(3HV)] and copolymer P(3HB-co-3HV). Among mcl-PHAs are homopolymers poly(3-hydroxyhexanoate) [P(3HHx)], poly(3-hydroxyoctanoate) [P(3HO)] and P(3HHx-co-3HO). The scl-PHAs consist of 3–5 carbon atoms, while mcl-PHAs have 6–15 carbon atoms (Rekhi et al. 2022). PHB is the most widely studied and best-characterised PHA which accumulates up to 80–90% of the cell dry weight (Anjum et al. 2016). The scl-PHAs are very stiff, brittle, and show high crystallinity (60–80%), while long-chain PHAs are flexible materials, characterised by a low degree of crystallinity (25%), and also have a low tensile strength, high elongation to break, low melting temperatures and a glass-transition temperature below room temperature (Anjum et al. 2016). The properties of PHAs, including, for example, flexibility, biodegradability and renewal, depend on the synthesis pathway, monomer composition and chemical structure (Kumar et al. 2020). The main general characteristics of PHAs are: water insolubility, resistance to hydrolytic degradation, resistance to ultraviolet radiation, poor acid and alkali resistance, chloroform solubility, biocompatibility (suitable for medical applications) and biodegradability. Bacterial PHAs, after appropriate purification, are non-toxic polymers. However, it should be remembered that the purification processes may cause losses in the efficiency of the PHA obtained (Muneer et al. 2020). Moreover, PHAs have natural piezoelectricity and impermeability properties. All these properties make them beneficial and sustainable plastics (Rekhi et al. 2022), with great application potential. The direction of their industrial application and market value depend on the properties of PHAs (molar mass, crystallinity, biodegradation rate, recyclability) (Anjum et al. 2016). scl-PHAs are most often used for the production of disposable items and packaging materials, while mcl-PHAs are suitable for high value-added applications, for example, medical applications: surgical sutures, implants and biodegradable drug delivery matrices (Kourmentza et al. 2017) (Fig. 2).

The unique features of PHAs make it possible to use these materials instead of traditional plastics in the medical, food, textile, agricultural industries and others. In the food industry, the main application direction of PHAs is packaging. From a packaging production point of view, the most important characteristics of PHAs are: thermoplasticity, insulation, water repellency and vapour barrier. Packaging materials made of PHAs can be in the form of films or strong thermoplastics. PHA packages protect the product against oxygen, water and CO_2_. In addition, they reduce the problem of loss of taste or the smell of the product (Rekhi et al. 2022). The food industry already uses products based on PHAs: cups, jars, containers, trays and disposable utensils for food packaging (Reichert et al. 2020; Rekhi et al. 2022). PHAs are also used in agriculture. One application is controlled-release mechanism in fertilisers. PHA-based fertilisers degrade in the soil, releasing their content over an extended period of time. This approach reduces labour and fertiliser costs. PHAs can also be used for the production of agricultural nets or grow bags — an environmentally friendly alternative to conventional polyethylene products (Rekhi et al. 2022). PHAs seem to be an attractive candidate for the production of quality materials (with good acoustic and thermal qualities) for the automotive industry (wheel covers, trim parts, interior panels, seat materials, tubing) (Mohanty et al. 2018). The broadly understood medical industry (medicine, biomedicine, tissue engineering, pharmacology) seems to be the largest potential market for PHAs. Potential biomedical applications of PHAs are drug delivery systems, 3D implants for bone tissue regeneration, regeneration of articular cartilage, wound healing, blood vessel reconstruction, artificial blood vessels, artificial heart tissues, organ reconstruction, artificial organ constructs, implants, scaffolds for tissue engineering and many others (Kourmentza et al. 2017; Mohanty et al. 2018; Chavan et al. 2021; Tan et al. 2021; Rekhi et al. 2022).

## PHAs synthesis/factors influencing PHAs synthesis

The synthesis of PHAs follows three pathways, depending on the bacterial strain and the type of carbon source. The first pathway for the synthesis of PHAs consists of three reactions. The first is catalysed by β-ketothiolase — the condensation of two acetyl-CoA groups to form acetoacetyl-CoA. This is followed by the reduction of acetoacetyl-CoA by NADPH dependent acetoacetyl-CoA dehydrogenase. Finally, poly(3-hydroxybutyrate) [P(3HB)] polymerises monomers (R)-3-hydroxybutyryl-CoA to P(3HB). Pathway II is mainly found in bacteria that use fatty acids as their primary carbon source. In the third pathway for PHA synthesis, various carbon sources can be used: sugars, oils and fatty acids (Anjum et al. 2016). The main factors influencing the synthesis of PHAs are: the type of microorganisms, the type of carbon source(s), the total carbon load, the nutrient concentration, the C/N and C/P ratios, the pH, the mode of fermentation and the fermenter operating parameters (Chavan et al. 2021; Rekhi et al. 2022). The concentration of trace metals (Fe, B, Cu, Mn, Mo, Zn) and the salinity of the culture medium also affect the efficiency of PHA synthesis (Rodriguez-Perez et al. 2018). The main components of the microbial medium for the production of PHAs by bacteria are carbon substrates such as glucose, sucrose, glycerol, organic acids (e.g. acetate) and oils (Anjum et al. 2016). For fatty substrates, the production efficiency of PHAs is about 0.6–0.8 g/g, while for sugars it is 0.3–0.4 g/g (Cruz et al. 2016). In addition to pure carbon sources, a number of different types of side-streams (corn, beet molasses, whey, residues from olive mills, waste fats) are also used to synthesise PHAs (Rodriguez-Perez et al. 2018). Only some industrial side-streams, which have an appropriate C/N/P ratio, can be applied without the supplementation of additional nutrients in the form of N and/or P sources, for example, cheese whey (Colombo et al. 2016) and some activated wastewater sludge (Morgan-Sagastume et al. 2014; Rodriguez-Perez et al. 2018). Increasing the C/N ratio promotes the accumulation of PHAs, while reducing the yield of cellular biomass (Raza et al. 2018). Phosphate concentration also plays an important role in the production of PHAs — the effectiveness of the microbiological production of PHA results from the limited availability of some nutrients in the medium, including phosphorus (Chavan et al. 2021). Optimisation of the carbon source concentration, C/N ratio, temperature and the pH of the culture and the speed of mixing increased the PHA content in the biomass of *Bacillus thermoamylovorans* PHA005 bacteria from 50.77 to 63.27% (Choonut et al. 2020, 2022).

## Industrial PHAs production

Plastics (petroleum-derived plastics, traditional mineral oil-based plastics) are an important element of our lives, used in households and in many industries (the food industry, healthcare, the pharmaceutical industry, the automotive industry, transport, construction and the electrical industry and electronics, the textile industry) due to their versatility, low price and functional properties (lightness, durability, resistance to degradation). It is estimated that in 2027, the value of the global plastics market will reach almost USD 580 billion. The main user of plastics is the packaging sector. Plastic has become one of the most important problems of the modern world — it is problematic for the environment due to the low degree of degradation. Plastics take around 100 years to completely degrade. Plastic makes our life, in many cases, more comfortable, but also negatively affects the environment and our health — the pollution of water bodies and soils, the ubiquitous microplastic (from 4.8 to 12.7 million tons of plastics made of fossil fuels goes into the oceans). Undoubtedly, we live in a time of crisis regarding the ubiquitous plastic (petrochemical origin), further research on new biodegradable polymers is ongoing. The solution may be PHAs (Anjum et al. 2016; Rekhi et al. 2022; Shen et al. 2022). PHAs, polylactic acid (PLA) and polybutylene succinate (PBS) are considered the green polymers of the future, which are to replace the commonly used plastics — polypropylene (PP) and low-density polyethylene (LDPE) (Kourmentza et al. 2017). PLA and PBS are formed as a result of polymerisation by, respectively, lactic acid and succinic acid. PHA is produced naturally as a result of the metabolic activity of various species of bacteria. The high production costs of PHAs will make it difficult to commercialise and popularise these biodegradable materials; they are more expensive compared to conventional polymers. The cost of producing PHB (one of the most studied PHAs) is 4–9 times higher than that of polyethylene. The price of PP is in the range of USD 0.60–0.87/lb (USD 1.33–1.81/kg), while for the biopolymer PHA it is USD 2.25–2.75 lb (USD 5.00–6.11 kg) (Kourmentza et al. 2017). According to other source, the price of 1 kg of PHA is €2.2–5.0 kg, USD 7.8–11.2 kg (Shen et al. 2022). The high cost of PHAs is largely due to the expensive culture media. Almost half of the production cost of PHAs is the carbon substrate (Kourmentza et al. 2017; Shen et al. 2022). PHA production can save an average of 2 kg of CO_2_ emissions per kg of PHA produced compared to the production of plastics from fossil fuels (Essel and Carus 2012). The industrialisation of PHAs would therefore be of great importance for the environment, especially if the production of PHAs is based on the disposal of industrial side-streams. To reduce the cost of obtaining PHAs, cheaper media, improved culture strategies and easier and more ecologically beneficial methods are needed to extract, purify and further process PHAs (Pagliano et al. 2021).

## PHAs production from side-streams

In recent years, there has been a lot of research into reducing the cost of producing PHAs. A large number of these publications focused on the valorisation of side-streams with the simultaneous production of PHAs. Current regulations and global trends (zero-waste policy, sustainable development) promote solutions for the environmentally friendly management of organic residues; hence, the design of bioprocesses for the management of side-streams for synthesising PHAs (Table 4). Industrial waste materials differ in terms of the content of individual components (carbon, nitrogen, phosphorus), so it is important to remember to select the appropriate strain for the disposal of a given side-stream, depending on its composition (Raza et al. 2018; Kumar et al. 2020; Monroy and Buitrón 2020; Brojanigo et al. 2022; Tyagi et al. 2022). A wide range of side-streams have been recycled under laboratory conditions for producing PHAs (its copolymers and terpolymers): industrial residues, spent/waste oils, lignocellulosic side-streams, agricultural and household waste materials, glycerine and waste sewage (effluent, wastewater) (Riedel et al. 2015; Anjum et al. 2016; Urbina et al. 2018; Rathika et al. 2019; Kadier et al. 2021).

One of the materials to watch out for is starch, a high molecular weight glucose homopolymer. Starch is the most important source of carbohydrates in the human diet, and is the most abundant polysaccharide in plants (Chi et al. 2021). Starch is composed of starch chains (amylose or amylopectin). The ratio of amylose to amylopectin varies depending on the botanical origin of the starch (Chi et al. 2021). Its rather complex structure limits the ability of microorganisms to use it as a carbon source, for example, in the production of PHAs. Not all bacteria have the ability to produce the amylolytic enzymes which are necessary to break down this polysaccharide (Raza et al. 2018). Waste starch from the agri-food industry can be directly transformed into PHB by specially selected bacteria (including *Azotobacter chroococcum*, some species of bacteria of the genus *Bacillus*) (Favaro et al. 2019). Thermophilic *Cupriavidus taiwanensis* bacteria, using corn starch, are able to produce PHA at a concentration of 2.1 g/L (Sheu et al. 2009). In turn, the bacteria *Bacillus megaterium* cultivated in a hydrolysed cassava starch by-product achieved a biomass yield of 4.7 g dry biomass/L — of which 29.7% was PHB (Krueger et al. 2012). According to Tian et al. (2022), *Photobacterium* TLY01 is able to produce poly(3-hydroxybutyrate-co-3-hydroxyvalerate) [(PHBV)] (4.01 g/L) using soybean oil, corn starch and valerate as components of the culture medium. For *Massilia* sp. UMI-21, the production of PHA from corn and soluble starch was, respectively: 1.20 and 0.90 g/L (Han et al. 2014). *Bacillus aryabhattai* T34-N4 is able to use hydrolysed cassava pulp and oil palm trunk starch to accumulate up to 17 wt% PHB of the cell dry weight (Bomrungnok et al. 2020).

Another side-stream that has been widely studied in terms of producing PHAs is molasses (sugarcane or beet molasses). Molasses is a waste from the production of sucrose; it is a solution from which it is no longer possible to crystallise sugar (Jamir et al. 2021). Molasses is an excellent source of essential ingredients for bacterial growth: sucrose, glucose, potassium salts, nitrogen (amino acids) and trace elements (calcium, magnesium and iron) (Favaro et al. 2019). There are also small amounts of vitamins in molasses (Jamir et al. 2021). The research presented by Gomaa (2014) showed that sugarcane molasses can be successfully used by bacteria as a component of the culture medium for the production of PHAs. The maximum production of PHA was recorded in 96 h of culture, both for *Bacillus subtilis* and *Escherichia coli*. The tested microorganisms produced PHA with an efficiency of 54.1% w/w (12.38 g/L) and 47.16% w/w (7.50 g/L), respectively. The growth and PHA yields were improved by supplementation of the molasses medium with 1% ethanol. *Methylobacterium* sp. ISTM1 bacteria produced 1.41 g PHA/L from molasses (9%) (Tyagi et al. 2022). Recombinant strains of *Cupriavidus necator* NCIMB 11599 (pKM212-SacC) and *Cupriavidus necator* 437–540 (pKM212-SacCLdhA) grown on an MR medium supplemented with sugarcane molasses produced 4.60 g P(3HB)/L and 0.58 g P(3HB-co-LA)/L, respectively (Jo et al. 2021). Kingsly et al. (2021) obtained 4.98 g PHA/L by culturing *Enterobacter cloacae* in sugarcane molasses (4%). For PHA synthesis, Shen et al. (2022) used a mixed microbial culture (MMC – obtained from various pig farms) and two types of organic wastewater (effluent from a sucrose acidified fermenter and a molasses waste acidified fermenter). Both waste materials are characterised by a high COD and a high concentration of volatile fatty acids (acetic acid, PA, butyric acid). The medium was composed of sucrose acidified fermenter effluent and molasses waste acidified fermenter effluent in a 1:1 ratio. As a result of these cultures, it was possible to obtain PHA with an efficiency of 26.88%. The addition of pyruvate (1 g) in a sequencing batch reactor (SBR) increased the productivity of PHA yielded to 53.58 g PHA/g VSS (volatile suspended solids) (%).

Oil side-streams are by-products that pose a real threat to the natural environment. Their reprocessing and storage generate high costs; therefore, it is necessary to search for new methods of their valorisation. An example may be the biotechnological utilisation of this type of waste material with the simultaneous production of bacterial metabolites, for example, PHAs. There are many advantages to using oil side-streams, however, it should be emphasised that these substrates increase the complexity of the production process. Oils, due to their hydrophobicity, do not dissolve in the aqueous growth medium. Therefore, when using waste oils as a component of the medium, emulsions should be prepared and added to facilitate the availability of the substrate for the microorganisms used (Budde et al. 2011). It is worth noting that on the basis of stoichiometric estimates, 1 g of linoleic acid gives 1.38 g of PHB, while 1 h of glucose theoretically gives 0.48 g of PHB (Gutschmann et al. 2022). The main chemical constituents of vegetable oils are triacylglycerols (TAGs). To be able to use oils as a carbon source for the production of PHAs, microorganisms should be characterised by their ability to produce lipases. Such enzymes hydrolyse TAGs to fatty acids which can be further converted to PHAs (Talan et al. 2020). For Tufail et al. (2017), the maximum PHA production was achieved after 72 h of cultivation (37 °C, 100 rpm) using *Pseudomonas aeruginosa* bacteria and waste frying oil (23.7 g/L). Obruca et al. (2010) used waste rapeseed oil with the addition of propanol (a precursor of 3-hydroxyvalarete) for the production of poly(3-hydroxybutyrate-co-3-hydroxyvalerate). They obtained (fed-batch mode) biomass and PHA yields of 138 g/L and 105 g/L (76%), respectively. The yield coefficient was equal to 0.83 g PHA/g oil. The addition of propanol at a concentration of 1% (v/v) enhanced both biomass formation and PHA accumulation. Sangkharak and Prasertsan (2013) subjected *Bacillus licheniformis* bacteria to mutagenesis (UV and N-methyl-N′-nitro-N-nitrosoguanidine) to increase the efficiency of PHA production from palm oil mill effluent. The M2-12 strain was characterised by the highest specific growth rate (0.09/h), the highest biomass yield (22.24 g/L) and the most effective PHA production (19.55 g/L) in a medium consisting of 3% palm oil mill effluent (without supplementation with trace elements). PHA accumulation increased 3.18-fold compared to the wild-type strain. In addition to waste vegetable fats, solid waste animal fat was also used to produce PHA (Gutschmann et al. 2023). The *Cupriavidus necator* (formerly *Ralstonia eutropha*) Re2058/pCB113 strain used produced 45 g PHA/L (a double-jacket feeding system was created to thermally liquefy the waste animal fat to employ a continuous feeding strategy). De Meneses et al. (2020) used the bacteria *Pseudomonas chlororaphis* subsp. *aurantiaca* DSM 19603 and glycerol as the sole carbon source. The maximum yield of biomass was 11.79 g d.w./L, mcl-PHA was 19wt% (2.23 g/L);these results show the prospect of valorisation of waste glycerine for producing PHAs.

Whey is also of great interest as a residue that can enable the cheaper production of PHAs. The main components of whey are lactose, proteins and lactic acid (Chourasia et al. 2022). Chang et al. (2021) used cheese whey (CW) that contains low amounts of lactose, they carried out a two-stage PHA production. In the first stage, the carbon sources present in the CW were converted into acetic acid by the bacterium *Acetobacter pasteurianus* C1. In the second step, the acetic acid produced earlier was converted into PHA by *Bacillus* sp. CYR-1 bacteria. The CYR-1 strain produced 240.6 mg PHA/L from tenfold diluted whey (5.7 g acetic acid/L), while the culture with fourfold diluted whey (12.3 g acetic acid/L) produced 126 mg PHA/L. Removal of the excess protein present in the CW increased the production of PHA to 411 mg/L. Khattab et al. (2021), using cheese whey (50%) and 1 g of ammonium chloride/L, produced 0.95 g PHA/L (20.96%, w/w) (*Bacillus flexus*). Whey as a carbon source was also used by Pais et al. (2016). Batch bioreactor cultivation of *Haloferax mediterranei* gave a biomass yield of 7.54 g/L with a polymer P(3HB-co-3HV) content of 53% (7.92 g/L). It can be concluded that side-streams of industrial technological processes may be promising raw materials for the production of PHAs. It should be noted that many research centres are still conducting intensive research work for developing a fast, efficient and environmentally friendly production process for PHAs, so as to increase the profitability of obtaining this metabolite, and, thus, its wider industrial use.

## Conclusions

This article shows that BC, PA, vitamin B12 and PHAs, having significant application potential, can be successfully produced with the use of bacteria and industrial side-streams. A number of different types of residues were used to obtain these compounds: lignocellulosic biomass, side-streams from the production of beverages, waste materials from the agri-food industry, textiles residues, food waste materials, side-streams from the fuel industry and others. Increasing comercialisation of the microbial production of the above compounds, by reducing production costs, will certainly bring tangible benefits, in many aspects: reducing the problem of vitamin deficiency among various social groups, eliminating conventional plastics and materials subject to long-term degradation from the market (replacing them with biodegradable materials), replacing substances chemically synthesised (processes harmful to the environment) with fully natural compounds (striving for products with the so-called “clean labels”). All thanks to the utilisation of side-streams with the simultaneous acquisition of metabolites of various bacterial species. Biotechnological valorisation of industrial waste will benefit the environment, economy and community of our planet. However, it is still necessary to improve biotechnological processes and increase the efficiency of production of individual compounds, so that their production is more profitable and beneficial. New side-streams still should be looked for, possibly the cheapest, which would contain substances that stimulate the metabolism of individual microorganisms. To increase the profitability of the microbiological synthesis of the described metabolites, statistical optimisation methods can also be used. Strains should be screened for efficient metabolite production, and metabolic and genetic modifications are an option. An important issue in terms of optimising the production of individual compounds is also the improvement of culture methods — immobilisation, the design of bioreactors. So far, great progress has been made in the microbiological valorisation of side-streams, so it seems that commercialisation of these solutions is just a matter of time.

## Data Availability

Not applicable.
